# Multispectral photoacoustic microscopy and NIR-II fluorescence imaging of TREM2-positive microglia in Aβ-driven Alzheimer’s pathogenesis

**DOI:** 10.1016/j.fmre.2026.04.016

**Published:** 2026-05-02

**Authors:** Hsuan Lo, Shiying Li, Jiali Chen, Qi Zhou, Yang Qiu, Shaoheng Ma, Bo Yu, Tiancheng Gu, Liming Nie

**Affiliations:** a Guangdong Cardiovascular Institute, Guangdong Provincial People’s Hospital, Guangdong Academy of Medical Sciences, Guangzhou 510080, China; b Medical Research Institute, Guangdong Provincial People’s Hospital (Guangdong Academy of Medical Sciences), Southern Medical University, Guangzhou 510080, China; c School of Medicine, South China University of Technology, Guangzhou 510006, China

**Keywords:** Multi-wavelength photoacoustic imaging, TREM2-expressed microglia, NIR-II fluorescence imaging, Alzheimer’s disease, Cerebrovascular imaging

## Abstract

Monitoring microglial activation mediators remains challenging in neurodegeneration. Few imaging studies track amyloid-β (Aβ)-linked microglial dynamics across a wide field of view with high spatiotemporal resolution. Leveraging the triggering receptor expressed on myeloid cells 2 (TREM2), a pivotal Alzheimer’s disease (AD) biomarker that enhances Aβ clearance while suppressing neuroinflammation, we developed a dual-modal probe, TREM2-ICG, by conjugating a TREM2-specific antibody with indocyanine green (ICG), an FDA-approved dye, for robust *in vivo* photoacoustic and near-infrared-II (NIR-II) fluorescence imaging. Multi-wavelength photoacoustic microscopy imaged AD pathology at 532 nm (hemoglobin, vasculature), 559 nm (Aβ probe AOI987), and 780 nm (TREM2-ICG for peri‑plaque microglia). Time-resolved NIR-II imaging (30 frames/sec) tracked Aβ-oligomer-induced microglial displacement at ∼50 µm resolution, showing a rapid chemotaxis phenomenon. Immunofluorescence-verified TREM2-microglia plaque engagement demonstrates AD pathogenesis. Overall, our multiscale photoacoustic-fluorescence imaging resolved cortex-wide Aβ-microglial interactions, combining wide-field, high-speed, and deep-penetration to overcome confocal depth and two-photon field of view limits. This enabled *in vivo* tracking of microglial responses to Aβ, revealing potential for investigating AD-specific mechanisms.

## Highlights

• Novel TREM2-ICG probe enables dual-modal photoacoustic/NIR-II imaging.
• *In vivo* tracking of microglial dynamics near Aβ plaques with neural context at 30 fps.
• Multiscale imaging overcomes depth/field-of-view limits of conventional microscopy.
• Mapping cortex-wide microglial chemotaxis toward neurotoxic Aβ oligomers in real-time.

## Graphical abstract

**Figure:**
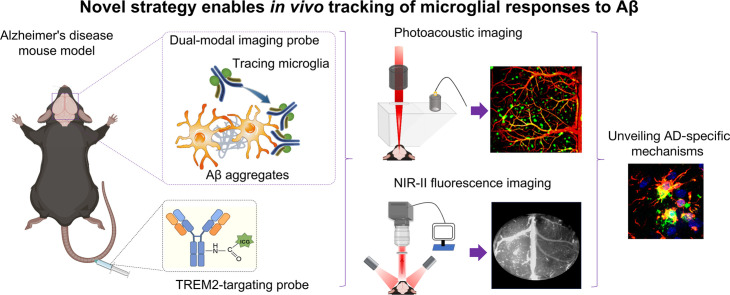

## 1 Introduction

Microglia serve as immune-capable macrophages of the brain, possessing a distinctive ability to monitor their immediate microenvironment and respond to tissue injury, infections, or disturbances to homeostasis [1]. Proper regulation of microglial activation mitigates pro-inflammatory cytokine release and attenuates chronic neuroinflammation, a key driver of neurodegenerative disorders, including Alzheimer’s disease (AD) [1]. Recently, the 18 kDa Translocator Protein (TSPO) has become a pivotal biomarker for *in vivo* quantification of microglial activation through positron emission tomography (PET), with over 300 clinical studies in the past decade establishing its utility in neuroinflammatory and neurodegenerative disease research [2,3]. While amyloid-beta (Aβ) plaque-induced neuroinflammation remains a hallmark pathological feature in AD pathogenesis, emerging comparative studies reveal critical interspecies limitations [4]. Human PET-TSPO reflects inflammatory cell density rather than activation states. This response profile diverges sharply from murine models, where TSPO expression increases in activated microglia, but remains unchanged in both non-human primate disease models and human neurodegenerative/neuroinflammatory conditions, demonstrating conserved interspecies limitations of TSPO as an activation marker [5]. This fundamental discrepancy underscores the need for next-generation imaging biomarkers and strategies with enhanced pathophysiological specificity. Notably, triggering receptor expressed on myeloid cells 2 (TREM2) represents a multifunctional therapeutic target in microglia, coordinating multiple neuroprotective mechanisms including dynamic modulation of microglial activation thresholds, Aβ aggregate containment through physical barrier formation, and enhanced phagocytic efficiency [6]. Thus, TREM2 emerges as a two-pronged target for both monitoring and treating AD-associated neuroinflammation. This potential is demonstrated in a recent study [7] through two key assessments. First, selective microglial enrichment leads to PET signal enhancement in amyloidosis mouse models. Second, human autoradiography reveals elevated tracer binding in the cortices of AD patients [7]. Emerging data further identify IgG-scFv as a promising new radioligand for *in vivo* advancing TREM2-targeted neuroinflammation studies and AD drug development [8]. Building on this, the synergistic integration of TREM2-targeted imaging with conventional Aβ plaque mapping enables multilayered monitoring of Aβ-induced neuroimmune cascades, thereby providing functional insights into early-stage immune dysregulation preceding clinical AD manifestations [9].

Increasingly, advanced optical imaging is now expected to provide whole-brain depictions with spatiotemporal resolution through engineered contrast agents, circumventing the radiation constraints inherent to current microglial imaging techniques such as TSPO-PET and conventional PET modalities [10]. Integrating brain imaging technologies to study physiological and vascular functions alongside molecular imaging significantly boosts possible research for AD [10]. As a forefront multimodal imaging platform, photoacoustic microscopy (PAM) uniquely combines optical excitation with ultrasonic detection, resolving both endogenous chromophores (hemoglobin, melanin) and exogenous molecular probes through laser-induced thermoelastic expansion. This hybrid mechanism enables non-invasive, multiscale imaging from subcellular (<5 µm resolution) to organ-level visualization, particularly excelling in simultaneous mapping of microvascular remodeling, including vessel density and oxygen saturation, via differential optical absorption spectra [11,12]. Recent advancements in high-resolution, real-time, and wide-field PAM have substantially enabled the progression of research into brain diseases, enhancing our capabilities in understanding and analyzing brain changes in detail [13,14]. Additionally, the integration of PAM in dual-wavelength modes with dyes and targeted probes has yielded significant progress in the realms of brain and lymphatic imaging in recent years [15]. Combining optical-resolution PAM (OR-PAM) with acoustic-resolution PAM (AR-PAM) enables high-resolution imaging that precisely delineates spatial resolution, providing in-depth visualization of meningeal lymphatic vessels and cerebral vasculature for neuroimaging applications [15]. Henceforth, the advantages of PAM allow for monitoring of pathological features in AD, which includes effectively observing immune cell responses to Aβ in the brain. This study aims to develop a method that integrates multi-wavelength PAM with microglia-targeted probes to investigate vascular architecture and brain cell function at multiple wavelengths.

Fluorescence imaging has emerged as a cornerstone in biomedical research due to its exceptional sensitivity and non-invasive nature, circumventing ionizing radiation risks associated with conventional modalities [16]. The recent advent of second near-infrared (NIR-II, 1000–1700 nm) imaging has redefined *in vivo* visualization, achieving tissue penetration depths surpassing visible light (400–700 nm) and NIR-I (700–900 nm) regimes by 3–5 folds [17]. However, conventional optical modalities face inherent physical barriers in whole-brain imaging. Confocal laser scanning microscopy (CLSM) is typically restricted to superficial imaging depths (<100 µm) due to strong tissue scattering [18]. While two-photon and multi-photon microscopy extends this limit, it is generally capped at ∼800 µm (layer IV of the cortex) and suffers from a narrow field-of-view (FOV, typically < 500 × 500 µm²), necessitating time-consuming stitching to visualize macroscopic networks [19,20]. Utilizing high-brightness and biocompatible fluorescent probes in NIR-II imaging effectively overcomes *in vivo* challenges like tissue absorption, autofluorescence, and photon scattering [21,22]. This technique offers deep tissue penetration, micron-scale spatial resolution, and a high signal-to-background ratio, enhancing diagnostic utility. Due to its advantages over traditional NIR-I fluorescence imaging, NIR-II bioimaging has become the preferred method for functional examinations in mice, including whole-body angiography, organ visualization, diagnosis, and imaging-guided tumor treatment [[23], [24], [25]]. The NIR-II fluorescence imaging has revolutionized neurovascular research through its unique combination of centimeter-scale field-of-view (FOV) and millimeter-level tissue penetration, enabling the transcranial visualization of cortical microvasculature (5–50 µm diameter) with exceptional spatial resolution (≤25 µm) [22]. This technique surpasses conventional modalities like two-photon excitation microscopy, particularly in overcoming its limited imaging depth and FOV constraints [26]. Through multispectral detection, NIR-II facilitates simultaneous mapping of vascular architecture and functional perfusion parameters, establishing itself as an indispensable tool for decoding neurovascular coupling mechanisms in preclinical AD models [22].

**Scheme 1:**
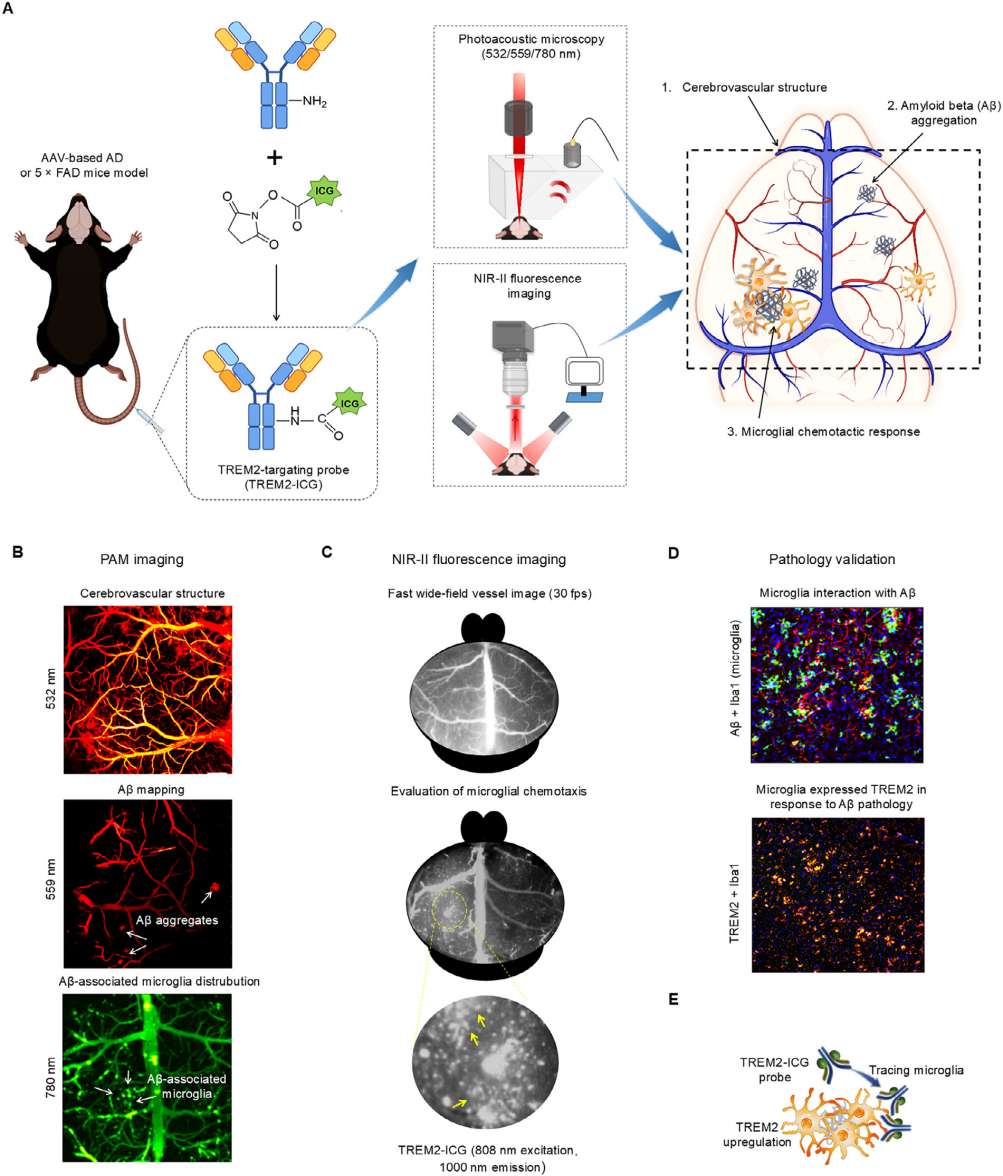
**Integrated schematic of dual-wavelength PAM and NIR-II fluorescence imaging for *in vivo* spatiotemporal mapping of Aβ-responsive microglial dynamics in Alzheimer’s disease.** (A) AD mouse models included (1) mice transfected via *in situ* AAV brain injection and (2) 5 × FAD transgenic mice. The exogenous contrast nanoprobe of TREM2-ICG was synthesized by conjugating NHS-ICG dye to TREM2 antibodies, which enhanced the visualization of reactive microglia. Dual-modal imaging, combining optical- and acoustic-resolution photoacoustic microscopy (OR/AR-PAM) with NIR-II fluorescence imaging, was utilized to distinctly visualize cerebral vasculature and activated microglia. (B-D) PAM/NIR-II fluorescence imaging was used to monitor cerebrovascular networks, Aβ pathology, and microglial motility in AD. Results were validated via immunofluorescent microscopy. (E) Schematic illustration of imaging AD-associated microglia using the TREM2-ICG probe, enabling effective monitoring of AD pathological progression.

In this study, we developed TREM2-ICG, a dual-modal probe engineered by conjugating clinical-grade indocyanine green (ICG) [27] with a humanized anti-TREM2 monoclonal antibody, leveraging the dual functions of TREM2 in enhancing Aβ clearance while suppressing neuroinflammation. Our imaging strategy combines multi-wavelength PAM and time-resolved NIR-II fluorescence imaging to achieve remarkable spatial and temporal resolution. The PAM system (532/559/780 nm excitation) provides comprehensive wide-field mapping at 5–40 µm resolution and 1.2 mm penetration depth, simultaneously resolving cerebral vasculature via endogenous hemoglobin contrast, localizing Aβ deposits, and detecting TREM2-positive microglial activation. This integrated approach reveals spatial relationships among vascular architecture, amyloid deposition, and TREM2-mediated neuroimmune responses (Scheme 1). Complementing this, our NIR-II fluorescence imaging achieves dynamic monitoring of Aβ-induced microglial directional migration with high temporal resolution (30 fps) and ∼50 µm spatial resolution, capturing rapid microglial repositioning towards stimulation sites within minutes - an early behavioral biomarker of neuroinflammatory cascades. ​This multiscale approach overcomes the inherent limitations of conventional microscopy (shallow penetration of confocal microscopy and narrow FOV of two-photon microscopy) by synergizing wide-field imaging, deep tissue penetration (>1 mm), and low phototoxicity. The system enables concurrent 3D reconstruction of cortical microvasculature (∼15 µm resolution at 532 nm) [28] and microglial distribution (via a 780 nm probe), along with presentation of morphologies that mimic confocal-observed microglial aggregates in size (∼70 µm diameter range, approximating native clusters). By visualizing sustained TREM2 expression dynamics in AD models and LPS-induced neuroinflammatory responses [29], our dual-modal platform provides novel insights into AD-specific pathogenic cascades, establishing a powerful tool for investigating microglia-mediated neuroimmune dysregulation during neurodegeneration.

## 2 Materials and methods

### 2.1 Chemicals

The fluorophore ICG-NHS ester was purchased from Xi’an Ruixi Biological Technology Co., Ltd. (China) and used without any further modification. Phosphate-buffered saline (PBS) and FITC—NHS were obtained from Sigma. AOI 987 is a near-infrared amyloid beta (Aβ) imaging probe purchased from MedChemExpress (USA). TREM2 antibody was purchased from Abcam (USA). Aβ_1–42_ oligomeric peptide was purchased from Biosensis (USA). LPS was purchased from Sigma (USA).

### 2.2 Synthesis and characterization of dual-imaging probe

As aforementioned [30], the TREM2 antibody was conjugated with ICG-NHS ester. To ensure optimal dual-modal performance, the conjugation stoichiometry was systematically optimized to achieve a specific dye-to-protein ratio (D/P). This optimized ratio balances the high quantum yield required for NIR-II fluorescence with the efficient photothermal conversion needed for photoacoustic imaging, thereby minimizing fluorescence quenching. Specifically, the antibody was incubated with the dye overnight at 4 °C. The resulting conjugate was purified using a 30 kDa Amicon^Ⓡ^ Ultra centrifugal filter (Millipore) to effectively remove unbound dye.

The dye-to-antibody ratio (DAR) was determined by measuring the absorbance of the purified conjugate at 280 nm (*A*_280_) and 780 nm (*A*_dye_) using a JASCO V-780 double-beam UV–Vis/NIR spectrophotometer. The DAR was calculated to be approximately 2.5 using the following formula:

(1) $$\text{DAR}=\frac{A_{\text{dye}}/\varepsilon_{\mathrm{dye}}}{\left(A_{280}-A_{\text{dye}}\times \text{CF}\right)/\varepsilon_{\text{IgG}}},$$

where ϵ_dye_= ∼22,884 L/mol*cm, ϵ_IgG_= ∼203,000 L/mol* cm (representing the extinction coefficient of the antibody), and the correction factor (CF) for ICG absorbance at 280 nm is 0.05 (adopted from a previously reported literature value) [31]. In addition, the absorption profile of the TREM2-ICG probe was further characterized across the 200–900 nm range.

### 2.3 Phantom test

To assess the penetration depth and resolution of NIR-I/II imaging, capillary glass tubes (*n* = 3 per group) containing TREM2-targeted probe dissolved in FBS were overlaid with chicken breast tissues of varying thicknesses: 0, 2.0, 4.0, 6.0, and 8.0 mm. NIR-II fluorescence images were subsequently obtained through the DPM-IVFM-NIR-II system (Zhuhai Dipu Medical Technology Co., Ltd). The correlation between tissue thickness and the resultant signal intensities or signal-to-background ratio (SBR) was analyzed in detail.

### 2.4 Microglia lines and cell culture

BV2 microglial cells, a murine microglial cell line, were maintained in Dulbecco’s Modified Eagle Medium (DMEM) supplemented with 10% heat-inactivated fetal bovine serum (FBS), 1% Penicillin/Streptomycin (P/S), and 4 mM L-Glutamine. Cells were cultured in a humidified incubator at 37 °C with 5% CO_2_. The culture medium was replaced every 2–3 days to ensure optimal growth conditions and to remove cellular debris. Upon reaching 80%–90% confluence, BV2 cells were passaged using 0.25% Trypsin-EDTA solution. After detachment, cells were centrifuged at 200 × *g* for 5 min and resuspended in a fresh, complete medium. All reagents were purchased from Gibco. Prior to conducting the PAM experiment, the cells were seeded in a culture dish and incubated with TREM2-ICG dye for 30 min. Following incubation, the cells were washed three times with PBS to eliminate any unbound dye.

### 2.5 Animal models

The 5 × FAD transgenic mice were generously provided by Professor Xiaodong Pan’s laboratory. These mice were originally acquired from The Jackson Laboratory (Stock #: 034840-Jax) and subsequently bred in-house with wild-type C57BL/6 mice. The 5 × FAD mice harbor human APP and PSEN1 transgenes that collectively comprise five mutations associated with AD: the Swedish (K670N/M671L), Florida (I716V), and London (V717I) mutations in the APP gene, along with the M146L and L286V mutations in the PSEN1 gene. Within the brain parenchyma, amyloid pathology initiates with the emergence of intracellular punctate amyloid-beta (Aβ) deposits, predominantly in the cortical and hippocampal regions. Thus, 5 × FAD mice at 8 months old (*n* = 3) were used for imaging and immunofluorescence assays to monitor the change in Aβ deposition and microglia. All animals were housed in a pathogen-free facility under a 12-h light/dark cycle, with environmental conditions carefully controlled to maintain a temperature of 21 ± 1.5 °C and humidity at 50 ± 10%. Additionally, all experiments adhered strictly to the Guidelines for the Care and Use of Research Animals and were approved by the Institutional Ethical Committee of Animal Experimentation of Guangdong Provincial People’s Hospital, China (permit number: KY2024-095-01).

### 2.6 Stereotaxic surgery for adeno-associated viruses (AAV) injection and LPS/Aβ aggregates induction

Male wild-type C57BL/6 mice, aged 8 weeks, underwent stereotaxic injections. Recombinant adeno-associated viruses (AAV2/9), pAAV-CMV-APP (K670N, M671L, I716V, V717I)-tWPA, and pAAV-CMV-PSEN1 (M146L, L286V)-tWPA (Heyuan) were mixed and bilaterally injected into the cortical region using a 10 µL Hamilton syringe with a 32G needle. In all experimental groups, the AAV was injected in a volume of 1 µL/side at a rate of 0.2 µL/min. The stereotaxic coordinates used were as follows: Anterior-Posterior (AP): +1.0 mm from Bregma, Medial-Lateral (ML): ±1.5 mm from the midline, and Dorsal-Ventral (DV): −1.2 mm. Antibiotics and analgesics were smeared on the surface of the wound of each mouse. Amyloid beta (Aβ) aggregates were prepared by dissolving Aβ_1–42_ peptides in deionized water to a concentration of 1 mg/mL. The samples were incubated in 0.5 mL closed siliconized plastic microcentrifuge tubes (Fisherbrand Low-Retention) on a Thermomixer (Eppendorf) set to 750 rpm at 22 °C for 48 h [32]. Lipopolysaccharide (LPS, 1 µL/mL) [33] and Aβ aggregates were injected into the cortex to induce pathological changes.

### 2.7 *In vivo* NIR-I fluorescence imaging of Aβ deposits in the mouse brain

The imaging method followed the procedures outlined in previous reports [34]. Briefly, for real-time observation of aggregated Aβ, mice received an intravenous injection of AOI-987 at a dose of 1 mg/kg in 0.9% saline. Near-infrared (NIR) fluorescence imaging of the brain was performed using the IVIS Spectrum *In Vivo* Imaging System (PerkinElmer, USA), with an excitation wavelength of 650 nm and an emission wavelength of 700 nm.

### 2.8 PAM imaging of the mouse brain

Mouse brain imaging employed a PAM system (Inno Laser Inc.) with triple excitation wavelengths (532/559/780 nm) and a 50 MHz ultrasonic transducer, achieving optical resolutions of 15 µm (532/559 nm) [28] and 70 µm (780 nm) at maximum penetration depths of 0.5 mm and 2 mm, respectively. For PAM imaging, mice were anesthetized using isoflurane (3.5% induction, 1.5%–2.0% maintenance) on a temperature-regulated platform. After creating a cranial window through midline incision and skull removal, we applied degassed ultrasound gel to the exposed cortex and secured the head in an imaging holder. Acoustic coupling was maintained using ultrapure water between the brain surface and the scanning membrane. The PAM system performed high-resolution imaging with 5 µm xy-step precision across large fields of view (3 × 3 mm² or 5 × 5 mm²). Multi-wavelength excitation was employed, with 532/559 nm targeting hemoglobin-based vascular contrast and 780 nm detecting exogenous probes. Following intravenous administration of Free-ICG and TREM2-ICG probes, dynamic imaging at 780 nm over a 5 mm FOV monitored probe distribution and microglial responses.

As previously described [28], data reconstruction in MATLAB 2021 performed vascular mapping (532/559 nm signals), microglial distribution analysis (780 nm signals), and microglial volumetric rendering. 3D Non-Local Means denoising and Hessian-based Jerman enhancement were applied for refinement of vascular features. HbO_2_ and Hb are the two major endogenous absorbers, which provide strong photoacoustic signals for oxygen saturation (*s*O₂) measurement. In essence, the blood absorption coefficient under two wavelengths can be expressed as follows [35]:

(2) $$\frac{\mu \left(\lambda_{1}\right)=\varepsilon_{\mathrm{Hb}}\left(\lambda_{1}\right)C_{\text{Hb}}+\varepsilon_{{\mathrm{HbO}}_{2}}\left(\lambda_{1}\right)C_{{\text{HbO}}_{2}}}{\mu \left(\lambda_{2}\right)=\varepsilon_{\mathrm{Hb}}\left(\lambda_{2}\right)C_{\text{Hb}}+\varepsilon_{{\mathrm{HbO}}_{2}}\left(\lambda_{2}\right)C_{{\text{HbO}}_{2}}}$$

In the above equations, $C_{Hb}$ and $C_{\mathrm{Hb}O_{2}}$ represent the Hb and HbO_2_ content, respectively, and $\varepsilon_{Hb}\left(\lambda_{1}\right)$, $\varepsilon_{Hb}\left(\lambda_{2}\right)$, $\varepsilon_{HbO_{2}}\left(\lambda_{1}\right)$ and $\varepsilon_{HbO_{2}}\left(\lambda_{2}\right)\;$ are the extinction coefficients of the Hb and HbO_2_ at wavelengths $\lambda_{1}$ and $\lambda_{2}$. Assuming the photoacoustic signal intensity is proportional to the absorption coefficient of the tissue, the sO_2_ can be calculated as follows:

(3) $$C_{\mathrm{Hb}}=\frac{\varepsilon_{{\mathrm{HbO}}_{2}}\left(\lambda_{2}\right)A\left(\lambda_{1}\right)-\varepsilon_{{\mathrm{HbO}}_{2}}\left(\lambda_{1}\right)A\left(\lambda_{2}\right)}{\varepsilon_{\mathrm{Hb}}\left(\lambda_{1}\right)\varepsilon_{{\mathrm{HbO}}_{2}}\left(\lambda_{2}\right)-\varepsilon_{\mathrm{Hb}}\left(\lambda_{2}\right)\varepsilon_{{\mathrm{HbO}}_{2}}\left(\lambda_{1}\right)}$$

(4) $$C_{HbO_{2}}=\frac{\varepsilon_{Hb}\left(\lambda_{1}\right)A\left(\lambda_{2}\right)-\varepsilon_{Hb}\left(\lambda_{2}\right)A\left(\lambda_{1}\right)}{\varepsilon_{Hb}\left(\lambda_{1}\right)\varepsilon_{HbO_{2}}\left(\lambda_{2}\right)-\varepsilon_{Hb}\left(\lambda_{2}\right)\varepsilon_{HbO_{2}}\left(\lambda_{1}\right)}$$

(5) $$\begin{array}{ccc} {\mathrm{sO}}_{2} & = & \frac{C_{{\mathrm{HbO}}_{2}}}{C_{\mathrm{Hb}}+C_{{\mathrm{HbO}}_{2}}}\\ & = & \frac{\varepsilon_{\mathrm{Hb}}\left(\lambda_{1}\right)A\left(\lambda_{2}\right)-\varepsilon_{\mathrm{Hb}}\left(\lambda_{2}\right)A\left(\lambda_{1}\right)}{A\left(\lambda_{1}\right)\left(\varepsilon_{{\mathrm{HbO}}_{2}}\left(\lambda_{2}\right)-\varepsilon_{\mathrm{Hb}}\left(\lambda_{2}\right)\right)+A\left(\lambda_{2}\right)\left(\varepsilon_{\mathrm{Hb}}\left(\lambda_{1}\right)-\varepsilon_{{\mathrm{HbO}}_{2}}\left(\lambda_{1}\right)\right)}\times 100\% \end{array}$$

In this equation, $A(\lambda_{1})$ and $A(\lambda_{2})$ represent the photoacoustic signal intensity obtained at wavelengths $\lambda_{1}$ and $\lambda_{2}$, respectively.

### 2.9 Second near-infrared (NIR-II) fluorescence imaging of the mouse brain

An electronic-cooling InGaAs short-wave infrared (SWIR) camera (640 × 512 pixels, Cheetah-640CL, Xenics) with a prime lens (focal length: 100 mm, antireflection coating at 800–1700 nm, Edmund Optics) cooled to −48 °C was employed to capture images in the NIR-II window. An 808 nm laser beam (Rayfine Specialty Lighting Co. Ltd, China) was expanded by a lens and connected to a collimator for uniform lighting across the field. Before each imaging session, the facular power density was measured and adjusted to 10–20 mW/cm². NIR-II fluorescence signals were detected using a 950 nm long-pass (LP) filter unless otherwise specified, with exposure times ranging from 50 to 500 ms. The NIR-II imaging FOV was approximately 10 cm.

### 2.10 Immunofluorescent staining

Preparation of the brain slices was described previously [36]. In brief, following a 1-h blocking step with 10% donkey serum (Sigma-Aldrich, USA), the slices were subsequently incubated with the primary antibodies of TREM2 (1:1000, Abcam, USA), Iba1 (1:1000, Abcam, USA), and Aβ (1:800, Abcam, USA) and then correspondingly incubated with the secondary antibody. DAPI was used as a counterstain for the cellular nuclei. ZEN microscopy software was used to quantify the proteins co-localized in microglia based on images acquired with the Zeiss LSM 980 Confocal Laser Scanning Microscope. Subsequently, the immunofluorescence intensities in the images were analyzed using Fiji software.

### 2.11 Statistical analysis

All quantitative data were analyzed and plotted using GraphPad Prism (version 9.0) and are presented as means ± standard error of the mean (SEM) in the graphs. Prior to statistical analysis, normality testing was conducted on the continuous variable data from each group. Quantitative data are reported as mean ± standard deviation (SD). The absorption and fluorescence emission spectra of the probe were normalized. Each group consisted of three mice for statistical analysis. Differences between groups were evaluated using two-tailed Student’s *t*-tests, with statistical significance indicated with *p < 0.05, **p < 0.01*,* and ***p < 0.001 considered statistically significant.

## 3 Results

### 3.1 Transcranial imaging of Aβ plaques

**Fig. 1:**
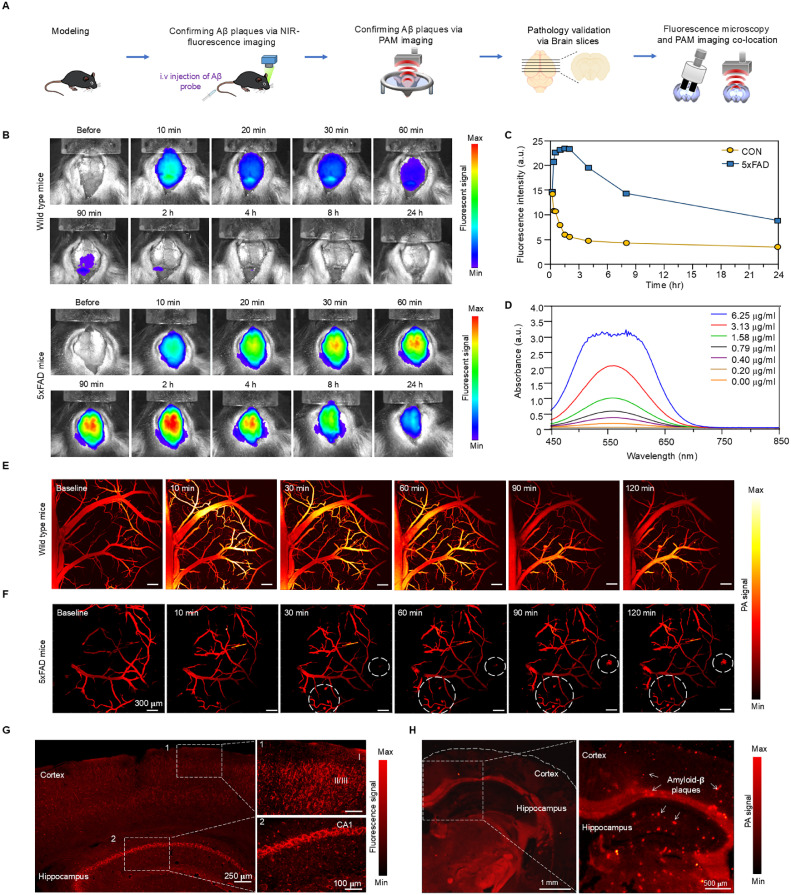
**Dual-modality *in vivo/ex vivo* imaging of amyloid-β plaque deposition in AD mouse models.** (A) Schematic illustration of the experimental design and workflow. (B) Representative NIR-I fluorescence images of brain Aβ distribution using the AOI-987 dye in wild-type (WT) and 5 × FAD mice over time. (C) Time-course quantification of brain fluorescence intensity in WT and 5 × FAD mice. (D) UV–Vis absorption spectra of the AOI-987 dye at various concentrations. (E-F) Longitudinal PAM imaging of the cerebral cortex in WT (E) and 5 × FAD (F) mice after probe injection (FOV: 3 mm × 3 mm, scale bar: 300 µm). (G) *Ex vivo* fluorescence microscopy images validating Aβ distribution in coronal brain sections. (H) Corresponding PAM imaging of Aβ plaques in coronal brain sections.

Fig. 1A provides a schematic overview of a series of experimental designs for imaging and verifying Aβ deposition in the mouse brain. This strategy combines NIR-I fluorescence imaging to indicate the overall expression level of Aβ. PAM imaging was used to determine the location of Aβ deposition, and brain tissue sections were analyzed with fluorescence microscopy and PAM to confirm the distribution of Aβ deposition in brain areas (Fig. 1A). First, we employed AOI-987 dye for NIR-I imaging by utilizing its ability to penetrate the blood-brain barrier (BBB) and specifically bind to Aβ plaques. As illustrated in Fig. 1B, from 0 to 120 min after tail vein injection of AOI-987, the fluorescent signal in the brain was significantly reduced in WT mice. In contrast, 5 × FAD mice retained a robust fluorescent signal, with intensity persisting for up to 24 h, indicating Aβ deposition in their brains (Fig. 1B). The fluorescence intensity analysis of the two mouse groups tracked changes in the AOI-987 dye over time and demonstrated that it persisted longer in the 5 × FAD mice (Fig. 1C). To establish the physical basis for subsequent applications, the *in vitro* absorbance of AOI-987 was measured. A primary absorption peak was identified in the 500–600 nm range (Fig. 1D). This absorption characteristic dictated the selection of the excitation wavelength for PAM imaging, ensuring high-sensitivity detection of Aβ plaques through strong photoacoustic signals (Fig. 1D). Before the AOI-987 injection, PAM imaging displayed the cerebral vascular structure of both WT and 5 × FAD mice (Fig. 1E,F). After administration, the PAM signal in WT mice increased significantly and gradually decreased after 60 min (Fig. 1E). In contrast, 5 × FAD mice showed speckle signals in extravascular structures from 30 to 120 min, highlighting the application of PAM imaging for visualizing Aβ deposition in the brain (Fig. 1F). A fluorescence microscope detected specific signals in the cortex and hippocampus, confirming the spatial distribution of Aβ in the brain (Fig. 1G). Interestingly, PAM imaging of 5 × FAD mouse brain slice offered a more three-dimensional reconstruction of Aβ plaque signals (approximately 500 µm thick) compared to fluorescent microscopy, with imaging sizes of 5 × 5 mm (Fig. 1H, left) and 3 × 3 mm (Fig. 1H, right). Interestingly, PAM enabled three-dimensional reconstruction of Aβ plaques across 500 µm-thick brain slices from 5 × FAD mice, providing large-field imaging over 5 × 5 mm^2^ and 3 × 3 mm^2^ fields of view (Fig. 1H) while preserving distribution details of plaque. This volumetric capability surpasses conventional fluorescence microscopy, overcoming its inherent limitations in depth penetration and optical sectioning for thick specimens. In the higher magnification 3 × 3 mm FOV, Aβ was notably observed in the cortex and hippocampus of 5 × FAD mice. These signals were absent in 5 × FAD mice without incubated dye and in WT mice incubated with dye (Fig. S1A,B). Furthermore, since PAM imaging is based on absorption values, these signals were different from the fluorescence signal caused by excitation light (Fig. 1G,H). Our findings demonstrate a strategy for imaging Aβ deposition in the brain across multiple scales. By utilizing an Aβ-specific dye, we highlight the advantages of PAM for evaluating Aβ deposition *in vivo*.

### 3.2 NIR-II TREM2-ICG probe synthesis and photoacoustic properties for microglial targeting

**Fig. 2:**
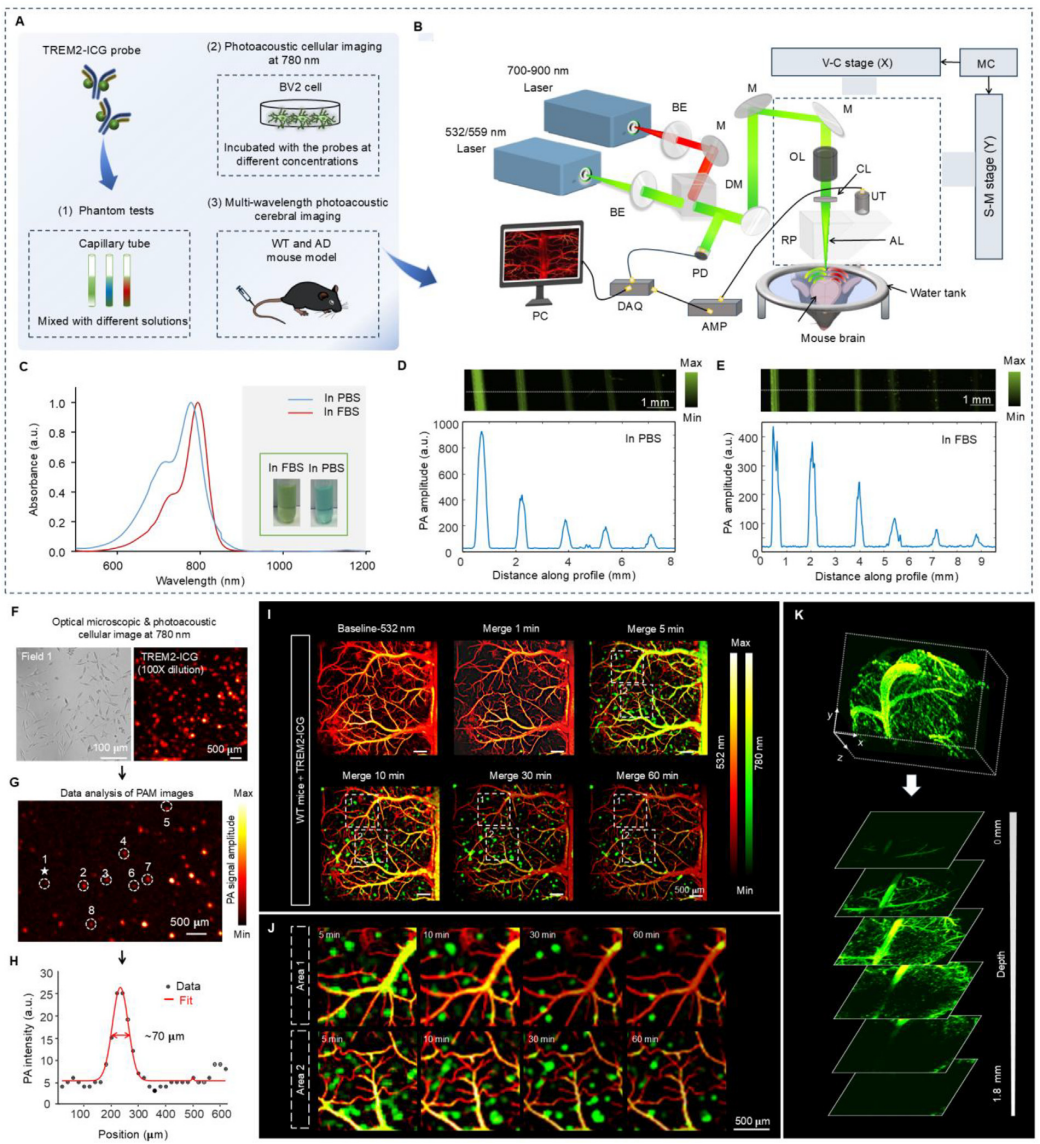
**Design and photoacoustic mapping of TREM2-targeted ICG probes.** (A) Schematic diagram of cell and animal experiments with the TREM2-ICG probe. (B) Schematic of the optical-resolution and acoustic-resolution photoacoustic microscopy (OR/AR-PAM) imaging system for brain imaging. BE, beam expander; M, mirror; DM, dichroic mirror; PD, Photodiode; OL, objective lens; CL, correction lens; UT, ultrasonic transducer; AL, acoustic lens; RP, rhomboid prism; MC, motion controller; Voice-coil stage (V-C stage); Stepper motor stage (S-M stage); AMP, amplifier; DAQ, data acquisition card; PC, personal computer. (C) Absorption spectra of the probe in PBS and FBS buffer. (D-E) The PA signal intensity of the probe dissolved in PBS and FBS at various concentrations (1.0, 0.5, 0.25, 0.125, and 0.0625 mg/mL). (F) Bright-field view of microglial cell line (BV2 cell, left). PAM imaging of BV2 cells with TREM2-ICG at 780 nm wavelength (right). Scale bar = 100 µm and 500 µm. (G-H) Quantitative full-width at half maximum (FWHM) profiling of cellular regions of interest (ROI) within the imaging field demonstrated spatial resolution characteristics, with mean FWHM measurements of 70.3 ± 2.1 µm (*n* = 8 cells) across the sampled microglial population. (I) Dual-wavelength PAM imaging shows the distribution of the TREM2-ICG probe over time and cerebral blood vessel signals. (J) Temporal changes of the enlarged PAM image from circle regions of interest (ROI) 1 and 2 in I. (K) Three-dimensional photoacoustic (PA) reconstruction enabled depth-resolved morphological mapping, demonstrating stratified tissue architecture across a 1.80 ± 0.03 mm z-axis penetration depth, achieved through multi-planar reconstruction.

TREM2, predominantly expressed by brain microglia, plays a critical role in regulating neuroinflammation and mediating phagocytic clearance of toxic protein aggregates in AD [37]. To enable microglial tracking through dual-modality imaging, we developed a NIR-II photoacoustic/fluorescence probe for microglial detection and integrated it with an OR/AR-PAM system for both *in vitro* and *in vivo* applications. As illustrated in Fig. 2A, the TREM2-ICG microglia-targeted probe was synthesized by conjugating the TREM2 antibody with ICG-NHS ester, following a previously reported method [38], with conjugation efficiency validated by BSA analysis (Fig. S2A). Subsequent biotoxicity assessments confirmed probe safety (Fig. S2B). The TREM2-ICG probe was then applied for: (1) phantom tests, (2) photoacoustic cellular imaging at 780 nm, and (3) multi-wavelength cerebral photoacoustic imaging (Fig. 2A). Using a multi-wavelength PAM platform equipped with discrete-wavelength lasers and integrated optical paths, we were able to simultaneously map both Aβ-associated microglia distribution via TREM2-ICG targeting and label-free cerebrovascular architecture (Fig. 2B). The photophysical properties of the TREM2-ICG conjugate were systematically characterized by NIR spectroscopy. A distinct bathochromic shift was observed in fetal bovine serum (FBS), with the absorption maximum shifting from 780 nm in phosphate-buffered saline (PBS) to 794 nm, corresponding to a 30 nm redshift. This spectral shift is attributed to J-aggregate formation induced by protein binding in the serum matrix (Fig. 2C). Fig. 2D,E display photoacoustic (PA) images of capillary glass tubes containing the TREM2-ICG probe dissolved in either fetal bovine serum (FBS) or phosphate-buffered saline (PBS) at concentrations ranging from 1.0 to 0.0625 mg/mL (1.0, 0.5, 0.25, 0.125, and 0.0625 mg/mL) in Fig. 2D,E. To assess the binding efficiency of the TREM2-targeted probe, we conducted correlative imaging analysis using (i) phase-contrast microscopy to examine the morphology and density of BV2 microglial cells prior to probe incubation, and (ii) PAM to evaluate probe binding at a 100 × dilution. The results demonstrate a concentration-dependent decrease in PAM signal intensity, indicating the probe’s specific binding affinity to TREM2-expressing microglia (Fig. 2F). In negative control experiments, free ICG showed complete signal loss after PBS washing (detection limit: <5 a.u., data not shown), demonstrating the necessity of TREM2 conjugation for cellular retention. After selecting regions of interest (ROIs) in BV2 cell PAM images, quantitative analysis revealed an average full width at half maximum (FWHM) of 70 ± 2.5 µm (*n* = 8 fields), demonstrating that the TREM2-targeted probe significantly enhances imaging specificity (Fig. 2H).

Next, the TREM2-targeted probe was administered to WT mice, followed by serial dual-wavelength PAM imaging. Reconstruction and overlay of the images revealed that the TREM2-targeted probe (green signal, 780 nm) localized primarily extravascularly, distinct from cerebral blood vessels (red signal, 532 nm) (Fig. 2I). Magnified views of the two ROIs allowed dynamic tracking of signal distribution, revealing the TREM2-ICG probe’s targeting pattern (Fig. 2J). Subsequent 3D reconstruction and analysis of PAM images mapped the spatial distribution of TREM2-ICG signals in the brain, achieving a depth penetration of 1.8 mm (Fig. 2K). Collectively, these results demonstrate that TREM2-targeted PAM imaging effectively distinguishes cerebral vasculature from TREM2-expressing cells, enabling 3D visualization of microglial distribution in the brain.

### 3.3 Visualizing microglial chemotactic dynamics with real-time NIR-II imaging *in vivo*

**Fig. 3:**
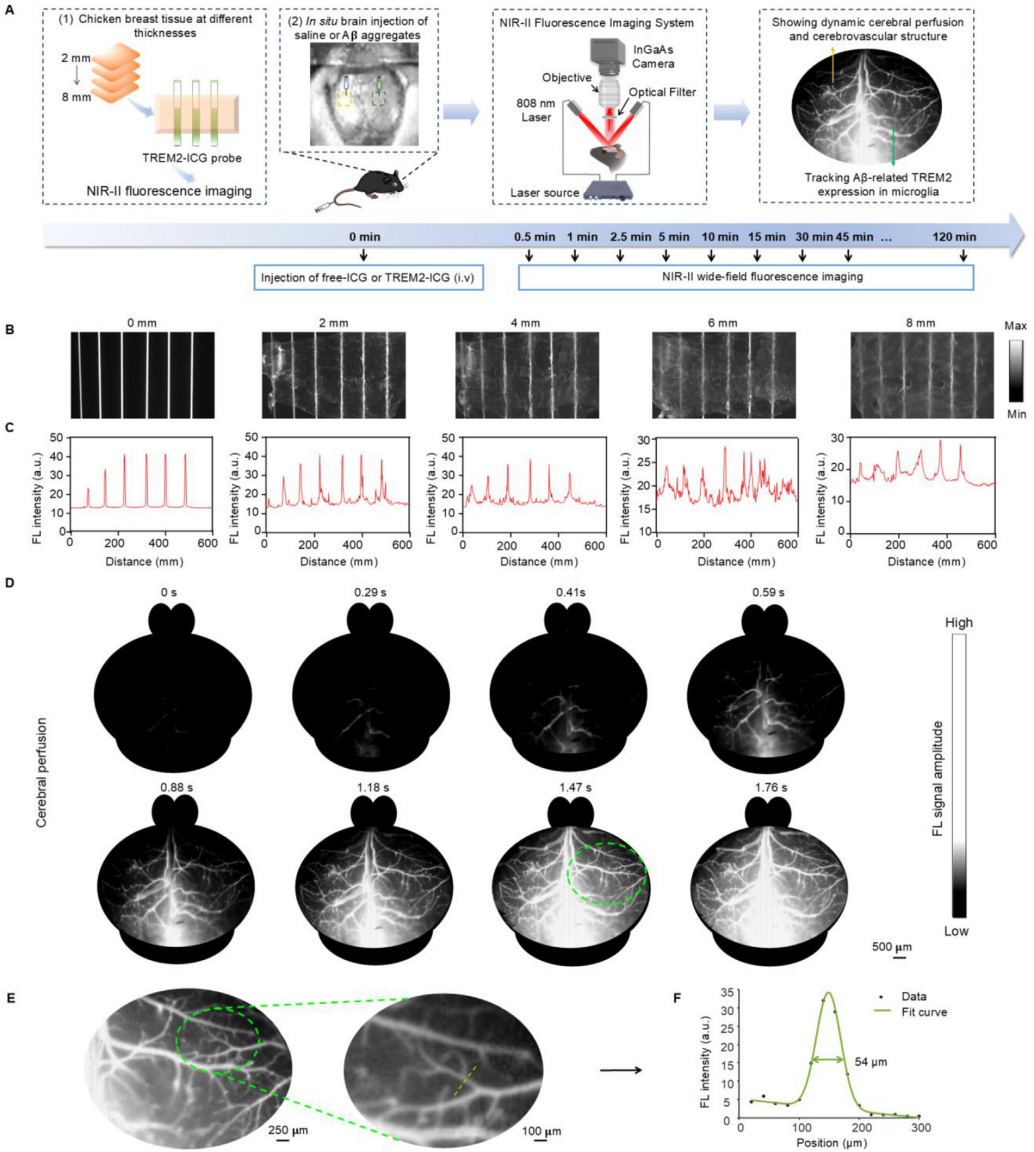
**NIR-II fluorescence characterization of TREM2-targeted *versus* free ICG probes.** (A) Strategic approach for tracking Aβ-associated microglial activation using NIR-II fluorescence brain imaging. (B) NIR-II fluorescence images of capillary glass tubes containing TREM2-targeted probes covered by chicken breast tissues of different thicknesses, namely 0, 2.0, 4.0, 6.0, and 8.0 mm (Exposure time = 200 ms, laser power = 5000 mW). (C) Fluorescence intensity profiles correspond to the NIR-II fluorescence image of (B). (D) Dynamic Free-ICG perfusion sequential images were captured with high temporal resolution within a 2-second window. (E) High-resolution detail of a selected ROI. Magnified view demonstrating the fine vascular structures resolved by the imaging system within the brain parenchyma. (F) The full width at FWHM of the blood vessel was 54 µm after the fluorescence cross-sectional distribution along the green dotted line in (E) and Gaussian fitting.

Amyloid plaque deposition induces a TREM2-dependent microglial phenotype transition (DAM-like state) characterized by altered chemotactic responses and injury susceptibility [39]. Capitalizing on the advantages of NIR-II imaging, including reduced light scattering and suppressed background signals, we established an internally controlled AD mouse model through bilateral intracerebral delivery of pre-aggregated Aβ_1–42_ oligomers (50 µM, 1 µL/hemisphere) *versus* saline controls to achieve high-contrast imaging and dynamic tracking of TREM2-expressing microglia (Fig. 3A). Using a NIR-II wide-field imaging system configured with dual optical fibers for uniform whole-brain illumination, we first evaluated the deep-tissue imaging performance of our TREM2-targeted ICG probe via NIR-II window fluorescence. Sequential NIR-II images were subsequently performed at defined intervals (0.5, 2, 3, 5, 10, 15, 30, 45, 60, 90, 120, and 150 min post-injection) to assess the spatiotemporal distribution dynamics of the probe in AD pathological mice (Fig. 3A).

Fig. 3B,C demonstrate the depth penetration capabilities of our TREM2-ICG probe through tissue-mimicking phantoms. Capillary glass tubes containing serially diluted probe solutions (0.5, 0.25, 0.125, 0.0625, and 0.03125 mg/mL in FBS) were imaged through increasing thicknesses of chicken breast tissue (2.0, 4.0, 6.0, and 8.0 mm). The NIR-II imaging system maintained excellent resolution up to 4.0 mm tissue depth, with all five concentrations clearly distinguishable (Fig. 3B,C). While some boundary blurring occurred at 6.0 mm, all samples remained detectable, and even at the maximum tested depth of 8.0 mm, probe localization was still possible (Fig. 3B,C). Comparative studies in PBS *versus* FBS revealed significant environmental dependence of probe fluorescence. Under 808 nm excitation in the NIR-II window (>1000 nm detection), the PBS-suspended probe showed minimal emission (signal-to-noise ratio < 2:1), while equivalent concentrations in FBS exhibited strong fluorescence (Fig. S2C,D). This serum-enhanced fluorescence suggests protein-binding-induced emission amplification, confirming the probe’s suitability for *in vivo* imaging applications.

**Fig. 4:**
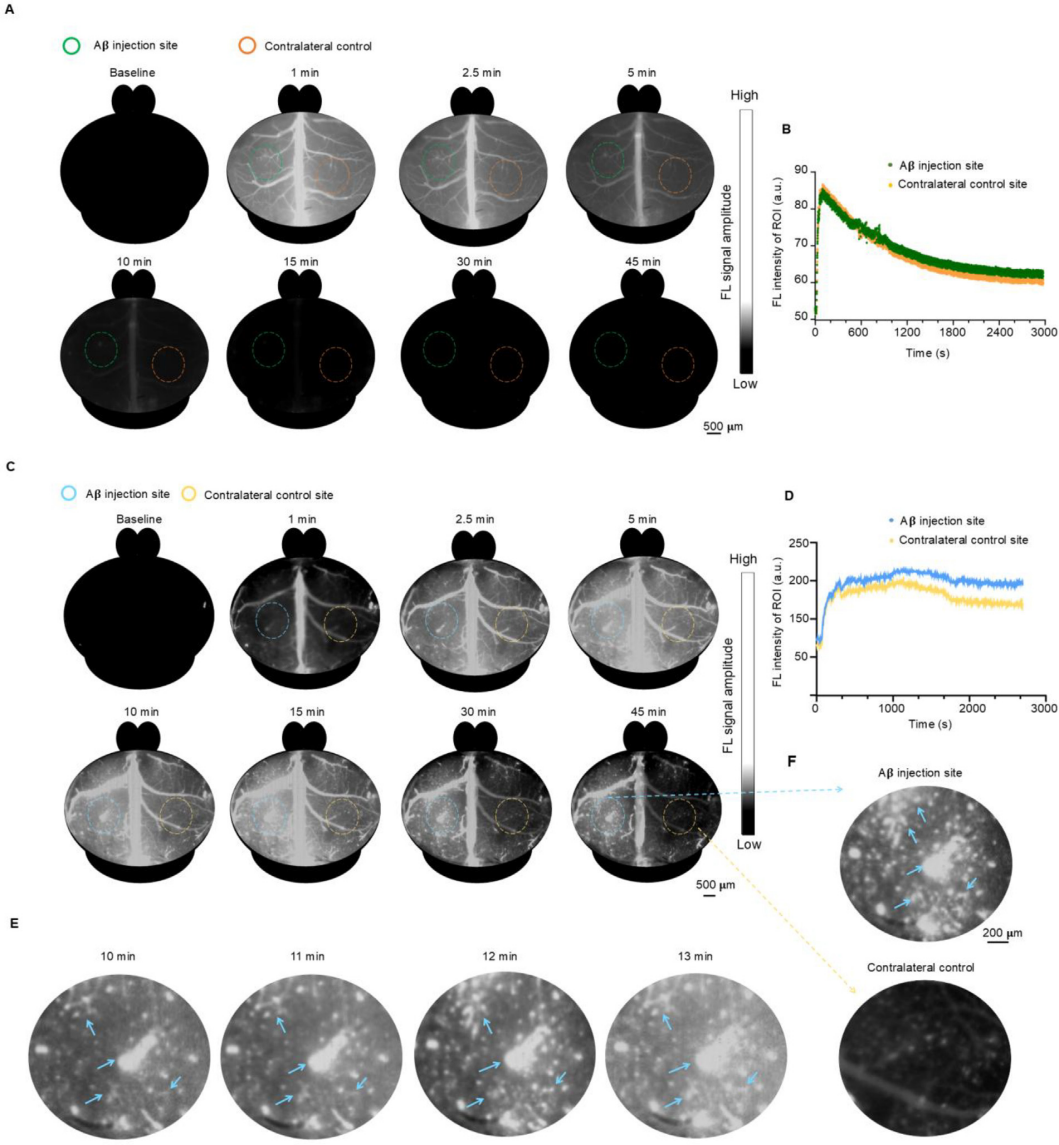
**Wide-field NIR-II imaging of microglial chemotaxis in Aβ pathology.** (A, C) NIR-II fluorescence images of targeted microglial distribution in the brains of Aβ aggregates-stimulated mice at 0.5, 1, 2, 3, 5, 10, 15, 30, 45 min following Free-ICG probe (A) and TREM2-ICG probe (C) injection. Scale bar: 500 µm. (B, D) Statistical plot showed the temporal measurements of fluorescence intensity within the ROIs corresponding to Aβ aggregates and saline injection sites in the brains of mice administered free-ICG (B) or TREM2-ICG probe (D). (E) Enlarged view at 10–13 min post-injection of TREM2 probe. Blue arrow: microglia. (F) Close-up view of Aβ injection site (left cortex) and contralateral control (right cortex) at 45 min post-injection. Scale bar: 200 µm.

Furthermore, we evaluated the time-resolved imaging capability of the NIR-II fluorescence system for brain perfusion analysis. As shown in Fig. 3D, whole-brain perfusion was achieved within 2 s using free ICG, revealing the spatial distribution pattern of cerebral blood flow at 0.1-s temporal resolution. The system successfully resolved fine structural details of the brain microvasculature across a wide field of view (Fig. 3D). Quantitative analysis of magnified ROI images demonstrated excellent signal-to-background ratios along with a spatial resolution of 54 µm for small cerebral vessels (Fig. 3E,F), enabling visualization of individual cell-sized structures. Building upon our validated imaging system, we performed *in vivo* tracking of microglial chemotaxis toward Aβ aggregates in an AD model. The TREM2-targeted probe showed significant signal accumulation in the Aβ-injected hemisphere relative to the saline-injected contralateral hemisphere (Fig. 4C), with high-resolution ROI images delineating these accumulation sites (Fig. 4F). Control experiments with free ICG revealed only background vascular patterns without specific binding (Fig. 4A). Importantly, free ICG exhibited minimal brain retention, with near-complete clearance observed within 30 min post-injection in both WT (Fig. S3) and Aβ-stimulated mice (Fig. 4A), thereby validating the targeting specificity of our TREM2 probe. To further evaluate the systemic biodistribution, we monitored the probe dynamics in major organs following i.v. injection. Dynamic dorsal imaging confirmed the gradual accumulation of ICG-TREM2 in the brain (Fig. S4A). Furthermore, *ex vivo* analysis at 120 min post-injection revealed that, consistent with the typical elimination pathway of ICG, the probe was primarily metabolized via the liver, while showing minimal non-specific uptake in the heart, spleen, lungs, and kidneys (Fig. S4B,C). Time-course analysis of signal intensity further revealed distinct kinetic profiles: in AD model mice administered free ICG, signals in both hemispheres decayed rapidly, whereas in mice injected with the TREM2-targeted ICG probe, signal retention was prolonged. Notably, the Aβ-injected hemisphere showed higher signal intensity than the saline-injected control side (Fig. 4B,D). Short-term imaging with high temporal resolution captured dynamic microglial changes across a wide field of view (Fig. 4E). Compared to the control injection site, the Aβ-injected region displayed more pronounced microglial aggregation, as evident in the magnified ROI (Fig. 4F).​ These results collectively demonstrate the utility of wide-field NIR-II imaging for tracking microglial activation dynamics in response to pathological stimuli.

### 3.4 PA imaging reveals AD brain vasculature and microglial distribution

**Fig. 5:**
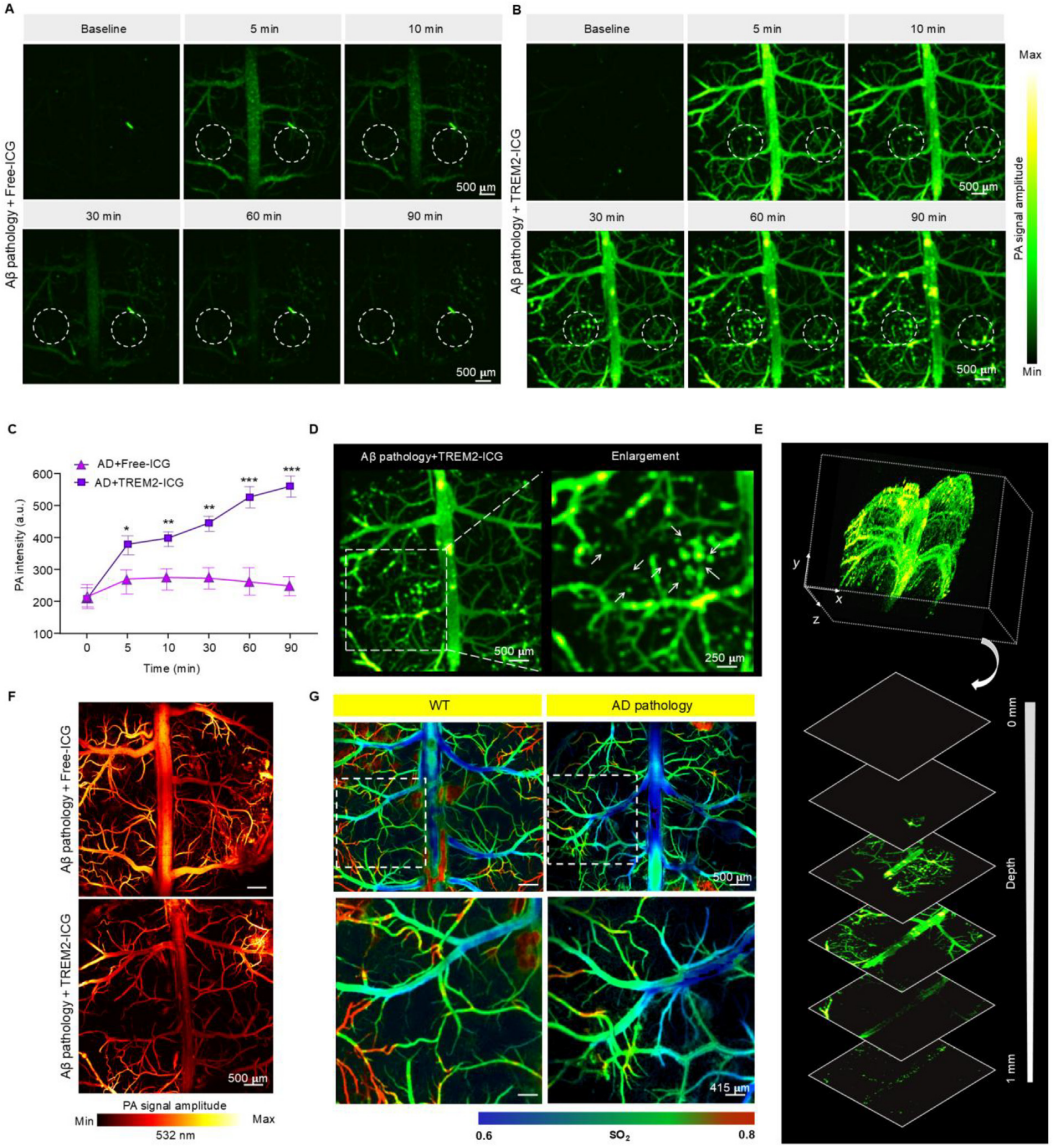
**Cerebral vasculature and microglia in AD by dual-wavelength PA imaging.** (A-B) Photoacoustic imaging of targeted microglial distribution in Aβ-stimulated mouse brains at 5, 10, 30, 60, and 90 min post-injection of (A) Free-ICG and (B) TREM2-ICG probes. (C) Quantitative analysis of PA signal intensity within ROIs (white dashed lines in A-B) over time. Data are presented as mean ± SD. *p < 0.05, **p < 0.01, ***p < 0.001 *vs.* AD + Free-ICG. *n* = 3. (D) Chronic cortical microglial accumulation revealed by TREM2-targeted PA signals in AD models. (E) Three-dimensional photoacoustic (PA) morphology reconstructed across a 1.0 mm depth range. (F) Photoacoustic cortical scans of cerebral vasculature in AD mouse models before administration of Free-ICG and TREM2-targeted probes. (G) Representative PA images of cerebral blood oxygen saturation (sO₂) in WT and AD mouse models acquired using dual-wavelength scanning.

PAM achieves high-resolution (axial: 15–50 µm; lateral: 5–20 µm) imaging of cerebral vasculature while quantifying hemoglobin oxygenation (sO₂) at 10% accuracy, leveraging hemoglobin as an endogenous contrast agent to simultaneously resolve capillary-level structural architecture and functional dynamics *in vivo* [40]. Capitalizing on this capability, we systematically investigated both microglial chemotactic responses and cerebrovascular dynamics under AD pathological conditions. Time-lapse PAM imaging quantitatively mapped spatiotemporal dynamics of TREM2^+^ microglial activation, revealing their coordinated migration patterns across the brain parenchyma (Fig. 5A,B). Under 780 nm excitation, PAM imaging with the TREM2-ICG probe selectively visualized amyloid-β-associated microglia through TREM2 targeting (Fig. 5A,B). Simultaneously, 532 nm excitation provided contrast-free imaging of cerebral vasculature (Fig. 5F). Quantitative analysis revealed sustained high-intensity PA signals in the TREM2-ICG group compared to rapid signal decay in free ICG controls (Fig. 5C), demonstrating specific probe retention in activated microglia. High-magnification PA imaging at 1-h post-injection clearly resolved extravascular signal distribution (Fig. 5D), demonstrating chronic microglial activation in AD pathology. In contrast, free ICG showed rapid brain penetration and clearance in control mice (Fig. S3), confirming the probe’s specificity. Additionally, to validate depth-resolved microglial mapping using NIR imaging, we performed 3D reconstruction of PAM images (Fig. 5E), successfully visualizing TREM2-ICG distribution throughout the brain with >1 mm penetration depth while maintaining microglial positional information.

Utilizing the photoacoustic spectral unmixing technique based on the distinct absorption spectra of oxyhemoglobin (HbO₂) and deoxyhemoglobin (HbR) in the visible range, simultaneous dual-wavelength PAM imaging (532/559 nm) revealed significantly reduced cerebral oxygen saturation (sO₂) in AD model mice compared to WT controls (*p* < 0.05, two-tailed *t*-test) (Fig. 5G and Fig S5). Regional sO₂ mapping demonstrated pronounced hypoxia (sO₂ = 0.6–0.7) in cortical microvasculature, indicating AD-associated microcirculatory dysfunction. In summary, we achieved the first simultaneous *in vivo* detection of cerebrovascular dynamics and neuroimmune responses to Aβ pathology in an AD mouse model using multi-wavelength PAM.

### 3.5 *In vivo* and *in vitro* evidence of LPS-induced microglia chemotaxis and microglial TREM2 expression in AD pathology

**Fig. 6:**
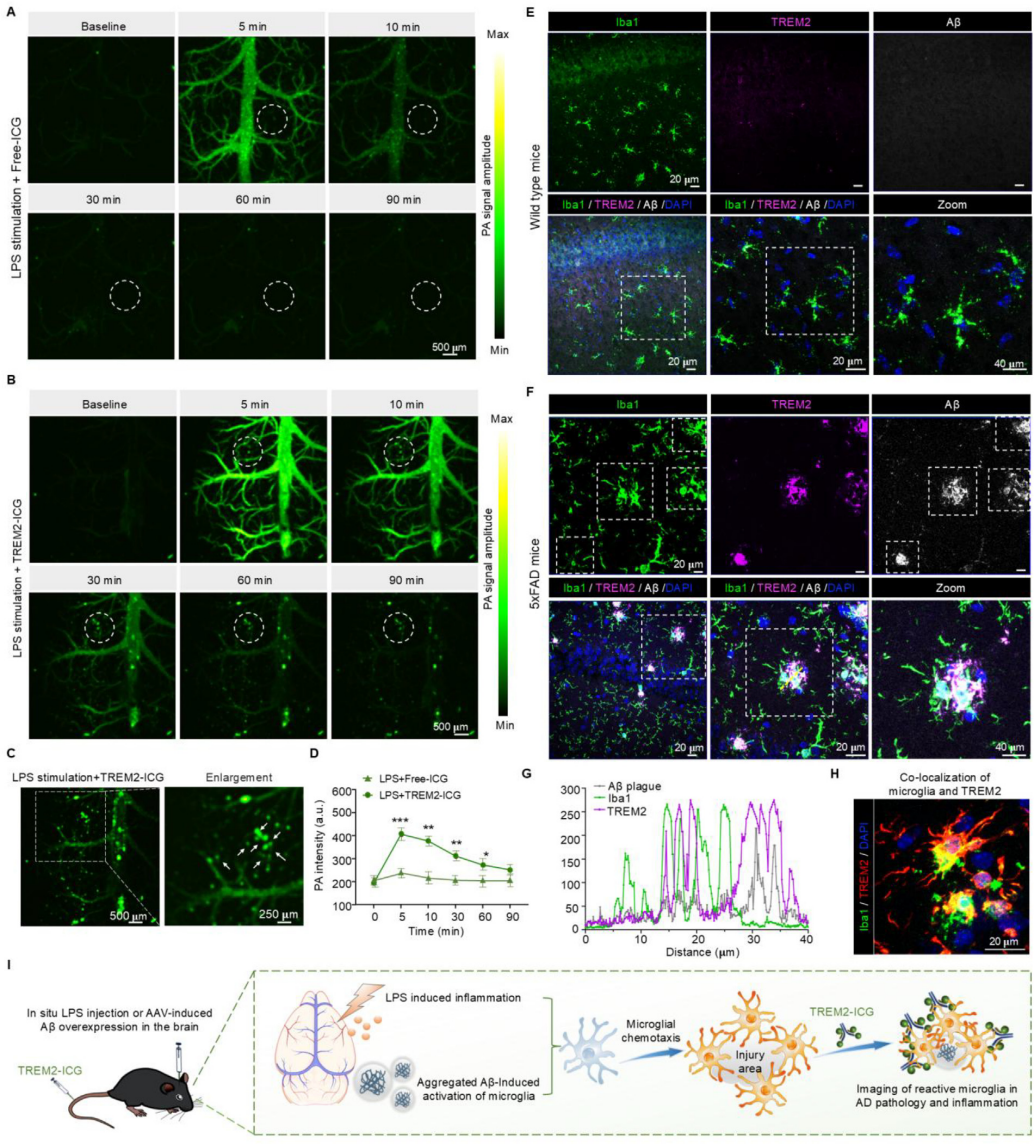
**PA imaging and immunofluorescence reveal LPS-induced microglia chemotaxis and microglial TREM2 expression in AD.** (A-B) Photoacoustic imaging of the cerebral cortex in LPS-induced mouse models after (A) Free-ICG and (B) TREM2-ICG probe injection. (C) Enlarged view of chronic extravascular microglial accumulation in the cortex visualized by TREM2-targeted photoacoustic imaging in LPS-induced neuroinflammation models. (D) Time-course quantification of PA signal intensity within ROIs (white dashed lines in A-B). Data are presented as mean ± SD. *p < 0.05, **p < 0.01, ***p < 0.001 *vs.* LPS + Free-ICG. *n* = 3. (E-F) Representative images showing triple immunofluorescence labeling of Iba1 (green, microglia), TREM2 (purple), and Aβ (white) in brain sections from WT (E) and 5 × FAD (F) mice. Scale bars are indicated for each magnification level. (G-H) Co-localization analysis (G) and high-magnification images showing TREM2 expression and microglial interactions with Aβ plaques. Magnification = 40×, scale bar = 20 µm. (I) Schematic illustration of LPS- and Aβ-induced microglia chemotaxis mapped via PA imaging with TREM2-targeted ICG probes.

Clinical studies demonstrate that superimposed systemic inflammation exacerbates AD progression [41]. Research has shown significantly upregulated TREM2 expression in LPS-stimulated primary microglia [42], consistent with its established anti-inflammatory role in mitigating LPS-induced neuroinflammation [43]. Based on this evidence, we intravenously administered TREM2-targeted ICG probes and performed real-time imaging to track microglial chemotaxis under LPS-induced neuroinflammatory conditions. As shown in Fig. 6A,B, administration of the TREM2-ICG probe generated a distinct retention signal, demonstrating its specific binding affinity for and tracking capability toward LPS-activated microglia. This selective labeling reliably identified inflammation-induced reactive microglial populations (Fig. 6C). In contrast, free ICG exhibited no detectable accumulation in LPS-stimulated brain parenchyma within 90 min post-perfusion (Fig. 6A). The time-dependent peak intensity of PA signals at the stimulation site demonstrated significantly higher retention of the targeted probe compared to free ICG, as quantified by longitudinal dwell-time analysis, establishing its sustained binding specificity *in vivo* (Fig. 6D). Leveraging the shared mechanism of TREM2 upregulation, we tailored the imaging strategy to distinct experimental objectives. While the AD model required wide-field NIR-II to track acute chemotaxis, the LPS model utilized a stereotactically defined focus to verify probe specificity. Since the goal was to confirm static target engagement within a predetermined ROI rather than monitor recruitment kinetics, we bypassed panoramic navigation and relied exclusively on depth-resolved PAM to validate precise 3D colocalization in the deep cortex.

Additionally, microglia exhibit a biphasic regulatory role in Aβ plaque dynamics, initially facilitating Aβ clearance before switching to a plaque-compacting phenotype [44]. During the compaction phase, TREM2-enriched microglial processes form dense perisomatic networks that encapsulate amyloid fibrils and nascent plaques, thereby promoting plaque densification and neuroprotective isolation [45]. In contrast, TREM2 deficiency disrupts this envelopment mechanism, leading to the formation of morphologically aberrant plaques characterized by elongated, highly branched fibrils. These structural abnormalities significantly increase the plaque surface area exposed to vulnerable neurites, exacerbating neuritic dystrophy [45]. To investigate this relationship, we performed immunofluorescence histochemistry to validate both Aβ plaque presence and TREM2 upregulation in AD mouse microglia. As shown in Fig. 6E,F, WT mice displayed minimal amyloid-beta deposition, scattered microglia distribution, and low TREM2 expression. In contrast, AD model mice exhibited numerous dense-core amyloid deposits encircled by microglia with significantly elevated TREM2 expression. Colocalization analysis revealed strong spatial overlap among Aβ plaques, microglia, and TREM2 signals (Fig. 6G,H). To further verify the probe’s ability to distinguish microglial states, we compared TREM2 expression in WT and 5 × FAD mice. Immunofluorescence confirmed that homeostatic microglia in WT brains exhibit minimal TREM2 immunoreactivity (Fig. 6E). Conversely, 5 × FAD brains displayed robust TREM2 upregulation specifically in plaque-associated microglia (Fig. 6F–H). This differential expression provides the molecular basis for the probe’s specificity, as the PA signal in AD models represents the recruitment of activated TREM2-positive microglia, whereas the lack of targets in WT mice results in minimal probe retention. These findings demonstrated that TREM2 upregulation specifically occurs in Aβ plaque-associated microglia, enabling them to form protective barriers around plaques and limit the spread of toxic Aβ species. Collectively, building upon two well-established neurobiological principles - TREM2’s role in Aβ plaque dynamics and its regulation of microglial inflammatory responses - the study illustrates a PAM imaging strategy that unites high-resolution wide-field detection with TREM2-targeted probes for microglial chemotaxis visualization (Fig. 6I).

## 4 Discussion

### 4.1 Clinically approved probe: compatibility and brightness

The clinically approved ICG used in our study is globally accessible in hospitals, offering superior cost-effectiveness and availability compared to other reported NIR-II fluorescent probes. Unlike carbon nanotubes, rare-earth-doped nanoparticles, quantum dots, and organic dyes, ICG effectively meets these practical requirements. Our findings demonstrate that ICG provides distinct optical advantages for murine and larger animal models. Although ICG is a well-established NIR-I fluorescent probe, it also exhibits significant NIR-II fluorescence emission [46,47]. ICG shows high absorbance, enabling effective NIR-II fluorescence bioimaging in mice. Notably, its absorbance in mouse blood is substantially higher in the 786–823 nm spectral range than in water. Furthermore, the NIR-II fluorescence signal of ICG in mouse blood was 5.2-fold stronger than in water. Under 808 nm laser excitation, ICG in mouse blood and fetal bovine serum (FBS) generated significantly brighter NIR-II emission, confirming its suitability for *in vivo* cerebral vasculature imaging in brain disease studies.

### 4.2 Pathology-facilitated brain delivery of the ICG-TREM2 probe

A critical consideration for antibody-based CNS probes is their ability to traverse the BBB. While monoclonal antibodies (∼150 kDa) generally exhibit restricted entry under physiological conditions, our study demonstrates effective brain parenchymal delivery of the ICG-TREM2 probe by leveraging disease-associated pathological changes. In Alzheimer’s disease and chronic neuroinflammation, BBB integrity is significantly compromised due to Aβ-induced downregulation of tight junction proteins and microvascular remodeling [[48], [49], [50]]. This pathological “leakiness” facilitates probe extravasation, a process analogous to the enhanced permeability and retention (EPR) effect typically observed in oncological imaging [51]. Our multi-wavelength PAM imaging provides direct visual evidence of this process, revealing ICG-TREM2 localization in the extravascular space, distinct from the cerebral vasculature (Fig. 2I,J). Furthermore, the sustained retention of ICG-TREM2 in Aβ-rich regions, compared to the rapid clearance of free ICG (Fig. 5A–C), underscores both successful extravasation and specific target binding to TREM2^+^ microglia. Beyond passive leakage, endogenous mechanisms such as adsorptive-mediated transcytosis (AMT) or specific transport systems may also contribute to the CNS entry of a small fraction of circulating antibodies [52,53]. The high sensitivity and superior background suppression of our NIR-II fluorescence and multi-wavelength PAM [54] systems enable high-contrast visualization of these probes even at low concentrations, facilitating the longitudinal tracking of microglial dynamics across the compromised BBB.

Following effective extravasation, accurately characterizing the probe’s parenchymal distribution requires distinguishing it from the dense vascular background. Here, multi-wavelength PAM serves as a “precision microscope” by employing a strategic spectral separation—utilizing 532/559 nm to map hemodynamic remodeling and 780 nm to isolate the ICG-TREM2 signal. This “system-probe matching” ensures that the delivered probe is spectrally distinct from endogenous hemoglobin, allowing for the precise depth-resolved reconstruction of its accumulation. Crucially, the retention of the probe within the parenchyma is dictated by biological specificity, where TREM2 acts as a functional anchor. Unlike constitutive markers such as Iba1, TREM2 is highly upregulated during neuroinflammatory activation. Consequently, the sustained PA signals observed in AD models (Fig. 5), in contrast to the transient vascular signals in homeostatic controls, confirm that the probe does not merely leak passively but actively binds to disease-associated microglia. This integration of spectral selectivity with biological specificity enables the volumetric quantification of Aβ-mediated microglial clustering relative to the neurovascular unit, providing 3D spatial insights into the delivery efficacy that planar NIR-II imaging cannot resolve.

### 4.3 Cellular specificity and probe bio-neutrality

It is important to clarify the cellular origin of our imaging signals. While TREM2 is a surface receptor expressed across various myeloid lineages, including both CNS-resident microglia and peripheral-derived macrophages, the signals captured by our TREM2-ICG probe in the 5 × FAD model predominantly reflect the resident microglial response. Previous lineage-tracing studies and single-cell transcriptomic analyses have demonstrated that the TREM2-positive cells encapsulating Aβ plaques are primarily derived from the local proliferation of resident microglia rather than the infiltration of peripheral monocytes [9,55]. This is consistent with our immunofluorescence results, where TREM2 expression was highly localized to Iba1+ cells exhibiting the characteristic morphology of disease-associated microglia (DAM). Although the BBB permeability in this model is sufficient for probe extravasation, it does not necessarily imply massive recruitment of peripheral cells. Consequently, while the TREM2-ICG probe serves as a marker for general TREM2-positive myeloid activation, in the context of AD-related neuroinflammation, it provides a reliable proxy for monitoring the resident microglial landscape. Moreover, binding specificity was rigorously validated through convergent lines of evidence: the probe exhibited sustained retention in AD mice compared to the rapid clearance of free ICG and the negligible background signals in wild-type controls. These *in vivo* findings, combined with our *in vitro* BV2 binding assays, strictly mirror the specific TREM2 upregulation confirmed by histological analyses, collectively ruling out non-specific off-target effects.

Beyond cellular specificity, ensuring the biological neutrality of the probe is paramount for accurate monitoring. A potential concern in antibody-based molecular imaging is the biological influence of the targeting moiety on its receptor. In this study, we purposefully employed a tracer-level dose of the TREM2 antibody, which is far below the threshold typically required for therapeutic modulation of microglial activity. This design minimizes the risk of the probe acting as an agonist or antagonist. Importantly, the robust chemotactic response observed in our NIR-II real-time imaging (Fig. 4) serves as an internal validation that the probe does not impair the fundamental ability of microglia to sense and migrate toward pathological stimuli. While the current study focuses on imaging efficacy, these findings suggest that the probe operates primarily as a reporter of the natural biological process rather than a functional perturber.

### 4.4 An innovative dual-modal imaging approach for hierarchical tracking of AD pathological progression

Current advanced imaging tools for investigating cellular dynamics in pathological brains face inherent trade-offs. Magnetic resonance imaging (MRI) provides whole-brain structural data but is fundamentally limited by insufficient spatiotemporal resolution (>100 µm) and an inability to monitor molecular-specific TREM2 dynamics at the cellular level. Multiphoton microscopy achieves cellular resolution with calcium imaging capacity, yet its clinical utility is constrained by photon scattering that limits penetration to depths of <1 mm, failing beyond cortical layers I/II. Similarly, positron emission tomography (PET) provides molecular tracking but cannot support longitudinal studies due to radiation exposure restrictions and minute-scale temporal resolution that preclude real-time monitoring.

Specifically, while high-resolution optical modalities like confocal and two-photon microscopy have been instrumental in AD research, they face inherent trade-offs when mapping brain-wide pathologies. Although our study employed a cranial window setup similar to these techniques, our strategic combination of PAM and NIR-II imaging overcomes specific limitations in scale and functional sensing. First, regarding imaging depth, confocal microscopy is typically restricted to a few hundred microns, and two-photon microscopy, while deeper, struggles to maintain high resolution beyond 600–800 µm due to photon scattering. In contrast, our PAM setup achieved a functional imaging depth of ∼1.8 mm (Fig. 2K), effectively capturing microglial distribution and vascular structures across the entire cortical thickness and reaching the upper hippocampus. Second, concerning the Field of View (FOV), standard two-photon systems often provide a restricted FOV (<1 mm²), making it difficult to monitor global immune responses. Conversely, our platform enables cortex-wide mapping (5 × 5 mm²), allowing for the quantification of long-distance microglial chemotaxis toward Aβ clusters that would be fragmented in narrower-field systems. Third, and most uniquely, our approach integrates metabolic information. Unlike fluorescence-only systems, our multi-wavelength PAM utilizes endogenous hemoglobin contrast to provide simultaneous oxygen saturation (sO₂) mapping (Fig. 5G). This allows us to correlate TREM2-positive microglial dynamics directly with the local microvascular environment, particularly in identifying hypoxic regions, providing a layer of functional insight that standard confocal or two-photon microscopy cannot readily provide. In comparison, photoacoustic platforms provide powerful structural-functional imaging. As demonstrated in our recent work, photoacoustic computed tomography (PACT) achieves unprecedented capabilities, including a large FOV (∼5 cm) with sustained high spatial resolution (∼110 µm at ≤2 mm depth) throughout deep brain structures (≤4 mm) [56]. Building on these foundations, this study establishes a novel differential diagnostic framework by capitalizing on the complementary strengths of PAM and NIR-II imaging. While currently performed on independent platforms, our strategy follows a “hierarchical imaging” logic, functionally coupled through the use of a single dual-modal TREM2-ICG probe and a multidirectional adjustable platform that ensures precise alignment for complete cranial window visualization. First, the approach leverages NIR-II fluorescence as a “global navigator.” We developed a customized NIR-II system (808 nm excitation with InGaAs detector) employing an area-excitation/area-detection strategy. This configuration minimizes tissue absorption and scattering, offering superior SBR and operational simplicity. Crucially, its wide FOV and high temporal resolution (30 fps) enable real-time screening of rapid microglial chemotaxis and simultaneous cortical angiography (Fig. 4), tasks that are impractical for point-scanning PAM due to inherent scanning latency. This step serves to identify optimal time windows and localize accumulation hotspots across the entire cortex. Following this broad screening, PAM functions as the high-resolution counterpart, characterizing the specific microglial microenvironment within these identified hotspots. By providing depth-resolved 3D structural mapping, this sequential integration allows for the volumetric quantification of Aβ-mediated microglial dynamics relative to the neurovascular unit—details that planar imaging cannot resolve. Collectively, this “macro-to-micro” workflow provides a comprehensive assessment that transcends the limitations of any single modality. By bridging the gap between whole-brain dynamic responses and capillary-level structural refinement, our platform establishes a robust, multiscale strategy for investigating the complex spatiotemporal progression of AD.

### 4.5 Limitations and future perspectives

First, regarding technical precision, our system utilizes a sequential scanning protocol (532/559 nm followed by 780 nm), introducing a temporal lag susceptible to physiological motion artifacts that can compromise micrometer-level registration. Furthermore, inherent wavelength-dependent resolution mismatches (∼15 µm at visible *vs.* ∼70 µm at NIR wavelengths) create ambiguity when defining precise boundaries at the *in vivo* microglia-plaque interface. Consequently, we employed multi-channel confocal immunofluorescence as the gold standard to validate these biological interactions. Future hardware upgrades, including achromatic optics and high-speed pulse-switching lasers, will be critical to bridge this resolution gap for real-time, coupled monitoring.

Second, regarding experimental rigor and specificity, while this study was designed as a proof-of-concept with a focused cohort and lacked TREM2-knockout controls due to logistical constraints, the substantial signal disparity between AD models and controls yielded clear statistical significance. We ensured reliability through methodological triangulation—validating consistent findings across NIR-II, PAM, and histology. Moreover, the high consistency between our *in vitro* binding assays and the distinct *in vivo* signal retention observed exclusively in AD pathology provides robust evidence that the imaging signals are actively mediated by specific antibody-antigen interactions rather than passive accumulation.

Third, regarding translational potential, we prioritized an open cranial window to maximize spatiotemporal resolution for tracking fine-scale chemotaxis. However, previous literature establishes that NIR-II fluorescence and acoustic-resolution PAM possess the capability to penetrate the intact mouse skull due to reduced scattering coefficients in the second near-infrared window [13,26]. While our current tissue-mimicking validations focused on intra-parenchymal detection, future studies aiming for non-invasive transcranial applications would benefit from hydroxyapatite-based simulations to precisely model skull attenuation and validate detection depths in a closed-skull setting.

## 5 Conclusion

This study pioneers the development of an ICG-conjugated TREM2 antibody probe for specific microglial labeling in AD. The probe enabled dual-modal NIR-II photoacoustic-fluorescent imaging, identifying Aβ plaque-associated microglia with excellent biocompatibility and selective brain accumulation. Spatiotemporal tracking of TREM2^+^ microglia via dual-wavelength PAM (∼70 µm resolution) was synergized with NIR-II fluorescence imaging for high-fidelity background suppression and dynamic scene sampling. This approach distinguished microglial activation patterns linked to Aβ pathology and correlated TREM2 expression with amyloid-beta deposition *in vivo*. Our platform offers significant potential for monitoring disease-specific microglial responses, advancing early AD detection, and pushing the frontiers for the development of precision therapies targeting AD mechanisms.

## CRediT authorship contribution statement

**Hsuan Lo:** Writing – original draft, Methodology, Data curation, Conceptualization. **Shiying Li:** Writing – original draft, Methodology, Investigation. **Jiali Chen:** Validation, Software, Data curation. **Qi Zhou:** Validation, Software. **Yang Qiu:** Software, Methodology. **Shaoheng Ma:** Validation, Data curation. **Bo Yu:** Writing – review & editing, Investigation. **Tiancheng Gu:** Visualization, Validation. **Liming Nie:** Writing – review & editing, Supervision, Resources, Project administration, Funding acquisition.

## Declaration of competing interest

The authors declare that they have no conflicts of interest in this work.

## Appendix Supplementary materials

Image, application 1

## References

[1] Gao C., Jiang J., Tan Y. (2023). Microglia in neurodegenerative diseases: mechanism and potential therapeutic targets. Signal. Transduct. Target. Ther..

[2] Guilarte T.R., Rodichkin A.N., McGlothan J.L. (2022). Imaging neuroinflammation with TSPO: a new perspective on the cellular sources and subcellular localization. Pharmacol. Ther..

[3] Zhang L., Hu K., Shao T. (2021). Recent developments on PET radiotracers for TSPO and their applications in neuroimaging. Acta. Pharm. Sin. B.

[4] Yenkoyan K.B., Kotova M.M., Apukhtin K.V. (2025). Experimental modeling of Alzheimer’s disease: translational lessons from cross-taxon analyses. Alzheimers Dement..

[5] Nutma E., Fancy N., Weinert M. (2023). Translocator protein is a marker of activated microglia in rodent models but not human neurodegenerative diseases. Nat. Commun..

[6] Hou J., Chen Y., Grajales-Reyes G. (2022). Trem2 dependent and independent functions of microglia in Alzheimer’s disease. Mol. Neurodegener..

[7] Shojaei M., Schaefer R., Schlepckow K. (2024). PET imaging of microglia in Alzheimer’s disease using copper-64 labeled TREM2 antibodies. Theranostics.

[8] Dahlén A.D., Roshanbin S., Aguilar X. (2025). PET imaging of TREM2 in amyloid-beta induced neuroinflammation. Eur. J. Nucl. Med. Mol. Imaging.

[9] Qi X., Zhu K., Ke W. (2025). Roles of TREM2 in Alzheimer’s disease. Transl. Neurodegener..

[10] Fang D., Wen X., Wang Y. (2022). Engineering of donor-acceptor-donor curcumin analogues as near-infrared fluorescent probes for in vivo imaging of amyloid-β species. Theranostics.

[11] Zhu X., Huang Q., DiSpirito A. (2022). Real-time whole-brain imaging of hemodynamics and oxygenation at micro-vessel resolution with ultrafast wide-field photoacoustic microscopy. Light Sci. Appl..

[12] Jiang S., Lin J., Huang P. (2023). Nanomaterials for NIR-II photoacoustic imaging. Adv. Healthc. Mater..

[13] Wang T., Chen J., Nie L. (2026). Photoacoustic microscopy for multiscale biological system visualization and clinical translation. Adv. Sci..

[14] Li H., Sun M., Zhu B. (2025). Multidimensional dynamic optical imaging unveils anesthetic-driven hemispheric lateralization in blood-brain barrier homeostasis. Sci. Adv..

[15] Yang F., Wang Z., Shi W. (2024). Advancing insights into in vivo meningeal lymphatic vessels with stereoscopic wide-field photoacoustic microscopy. Light. Sci. Appl..

[16] Hu Z., Fang C., Li B. (2020). First-in-human liver-tumour surgery guided by multispectral fluorescence imaging in the visible and near-infrared-I/II windows. Nat. Biomed. Eng..

[17] Zhou Q., Nozdriukhin D., Chen Z. (2022). Depth-resolved localization microangiography in the NIR-II window. Adv. Sci..

[18] Jiang T., Gong H., Yuan J. (2023). Whole-brain optical imaging: a powerful tool for precise brain mapping at the mesoscopic level. Neurosci. Bull..

[19] Qin Z., She Z., Chen C. (2022). Deep tissue multi-photon imaging using adaptive optics with direct focus sensing and shaping. Nat. Biotechnol..

[20] Zhao Z., Zhou Y., Liu B. (2023). Two-photon synthetic aperture microscopy for minimally invasive fast 3D imaging of native subcellular behaviors in deep tissue. Cell.

[21] Feng Z., Tang T., Wu T. (2021). Perfecting and extending the near-infrared imaging window. Light. Sci. Appl..

[22] Cai Z., Zhu L., Wang M. (2020). NIR-II fluorescence microscopic imaging of cortical vasculature in non-human primates. Theranostics.

[23] Li C., Chen G., Zhang Y. (2020). Advanced fluorescence imaging technology in the near-infrared-II window for biomedical applications. J. Am. Chem. Soc..

[24] Lei Z., Zhang F. (2021). Molecular engineering of NIR-II fluorophores for improved biomedical detection. Angew. Chem. Int. Ed. Engl..

[25] Yang Y., Xie Y., Zhang F. (2023). Second near-infrared window fluorescence nanoprobes for deep-tissue in vivo multiplexed bioimaging. Adv. Drug. Deliv. Rev..

[26] Wang F., Zhong Y., Bruns O. (2024). In vivo NIR-II fluorescence imaging for biology and medicine. Nat. Photonics..

[27] Szpunar M., Aebisher D., Wal A. (2025). Changes in absorption spectra of indocyanine green after visible light exposure and cold dark storage. Spectrochim. Acta. A Mol. Biomol. Spectrosc..

[28] Qin W., Li H., Chen J. (2025). Amphiphilic hemicyanine molecular probes crossing the blood-brain barrier for intracranial optical imaging of glioblastoma. Sci. Adv..

[29] Ohm M., Hosseini S., Lonnemann N. (2024). The potential therapeutic role of itaconate and mesaconate on the detrimental effects of LPS-induced neuroinflammation in the brain. J. Neuroinflammation..

[30] Chen J., Li S., Zhou Q. (2025). Near-infrared II fluorescence imaging highlights tumor angiogenesis in hepatocellular carcinoma with a VEGFR-targeted probe. Small. Methods.

[31] Wilson K.E., Bachawal S.V., Abou-Elkacem L. (2017). Spectroscopic photoacoustic molecular imaging of breast cancer using a B7-H3-targeted ICG contrast agent. Theranostics.

[32] Yusko E.C., Prangkio P., Sept D. (2012). Single-particle characterization of Aβ oligomers in solution. ACS Nano.

[33] Shivling Mali A., Honc O., Hejnova L. (2023). Opioids alleviate oxidative stress via the Nrf2/HO-1 pathway in LPS-stimulated microglia. Int. J. Mol. Sci..

[34] Zeng F., Fan Z., Li S. (2023). Tumor microenvironment activated photoacoustic-fluorescence bimodal nanoprobe for precise chemo-immunotherapy and immune response tracing of glioblastoma. ACS Nano.

[35] Cao F., Qiu Z., Li H. (2017). Photoacoustic imaging in oxygen detection. Appl. Sci..

[36] Feng L., Lo H., Zheng J. (2024). Cycloastragenol reduces microglial NLRP3 inflammasome activation in Parkinson’s disease models by promoting autophagy and reducing Scrib-driven ROS. Phytomedicine.

[37] Ulrich J.D., Ulland T.K., Colonna M. (2017). Elucidating the role of TREM2 in Alzheimer’s disease. Neuron.

[38] Carr J.A., Franke D., Caram J.R. (2018). Shortwave infrared fluorescence imaging with the clinically approved near-infrared dye indocyanine green. Proc. Natl. Acad. Sci. U. S. A..

[39] Wang Y., Cella M., Mallinson K. (2015). TREM2 lipid sensing sustains the microglial response in an Alzheimer’s disease model. Cell.

[40] Liu C., Wang L. (2022). Functional photoacoustic microscopy of hemodynamics: a review. Biomed. Eng. Lett..

[41] Xie J., Van Hoecke L., Vandenbroucke R.E. (2021). The impact of systemic inflammation on Alzheimer’s disease pathology. Front. Immunol..

[42] Wang M., Gao X., Zhao K. (2019). Effect of TREM2 on release of inflammatory factor from LPS-stimulated microglia and its possible mechanism. Ann. Clin. Lab. Sci..

[43] Huang P., Zhang Z., Zhang P. (2024). TREM2 deficiency aggravates NLRP3 inflammasome activation and pyroptosis in MPTP-induced Parkinson’s disease mice and LPS-induced BV2 cells. Mol. Neurobiol..

[44] Casali B.T., MacPherson K.P., Reed-Geaghan E.G. (2020). Microglia depletion rapidly and reversibly alters amyloid pathology by modification of plaque compaction and morphologies. Neurobiol. Dis..

[45] Yuan P., Condello C., Keene C.D. (2016). TREM2 haplodeficiency in mice and humans impairs the microglia barrier function leading to decreased amyloid compaction and severe axonal dystrophy. Neuron.

[46] Wu D., Xue D., Zhou J. (2020). Extrahepatic cholangiography in near-infrared ii window with the clinically approved fluorescence agent indocyanine green: a promising imaging technology for intraoperative diagnosis. Theranostics.

[47] Bhavane R., Starosolski Z., Stupin I. (2018). NIR-II fluorescence imaging using indocyanine green nanoparticles. Sci. Rep..

[48] Sweeney M.D., Sagare A.P., Zlokovic B.V. (2018). Blood-brain barrier breakdown in Alzheimer disease and other neurodegenerative disorders. Nat. Rev. Neurol..

[49] Nehra G., Promsan S., Yubolphan R. (2024). Cognitive decline, Aβ pathology, and blood-brain barrier function in aged 5xfad mice. Fluids Barriers CNS.

[50] Yang Q., Yu J., Qin H. (2021). Irbesartan suppresses lipopolysaccharide (LPS)-induced blood-brain barrier (BBB) dysfunction by inhibiting the activation of MLCK/MLC. Int. Immunopharmacol..

[51] Izci M., Maksoudian C., Manshian B.B. (2021). The use of alternative strategies for enhanced nanoparticle delivery to solid tumors. Chem. Rev..

[52] Terstappen G.C., Meyer A.H., Bell R.D. (2021). Strategies for delivering therapeutics across the blood-brain barrier. Nat. Rev. Drug Discov..

[53] Sweeney M.D., Zhao Z., Montagne A. (2019). Blood-brain barrier: from physiology to disease and back. Physiol. Rev..

[54] Tsuchikama K., An Z. (2018). Antibody-drug conjugates: recent advances in conjugation and linker chemistries. Protein Cell.

[55] Park M.D., Silvin A., Ginhoux F. (2022). Macrophages in health and disease. Cell.

[56] Pang W., Zhu B., Li H. (2024). Direct monitoring of whole-brain electrodynamics via high-spatiotemporal-resolution photoacoustics with voltage-sensitive dye. Laser Photon. Rev..

