# Supplementary material for: Multispectral photoacoustic microscopy and NIR-II fluorescence imaging of TREM2-positive microglia in Aβ-driven Alzheimer’s pathogenesis
Source: Fundam Res. 2026 May 2;6(4):2235–49. doi: 10.1016/j.fmre.2026.04.016 (PMC13424169; doi:10.1016/j.fmre.2026.04.016)
Supplement: Supplementary file 1 [file mmc1.docx]

**Supporting Information**

**Multispectral Photoacoustic Microscopy and NIR-II Fluorescence Imaging of TREM2-Positive Microglia in Aβ-Driven Alzheimer’s Pathogenesis**

Hsuan Lo^a,b,1^, Shiying Li^a,b,1^, Jiali Chen^b,c^, Qi Zhou^a,b^, Yang Qiu^b^, Shaoheng Ma^b^, and Liming Nie^a,b,*^

a. Guangdong Cardiovascular Institute, Guangdong Provincial People's Hospital, Guangdong Academy of Medical Sciences, Guangzhou 510080, China

b. Medical Research Institute, Guangdong Provincial People's Hospital (Guangdong Academy of Medical Sciences), Southern Medical University, Guangzhou 510080, China.

c. School of Medicine, South China University of Technology, Guangzhou 510006, China

*Corresponding author: Liming Nie

Email: nieliming@gdph.org.cn; ORCID ID: orcid.org/0000-0002-1781-4612

^1^These authors contributed equally to this work

**
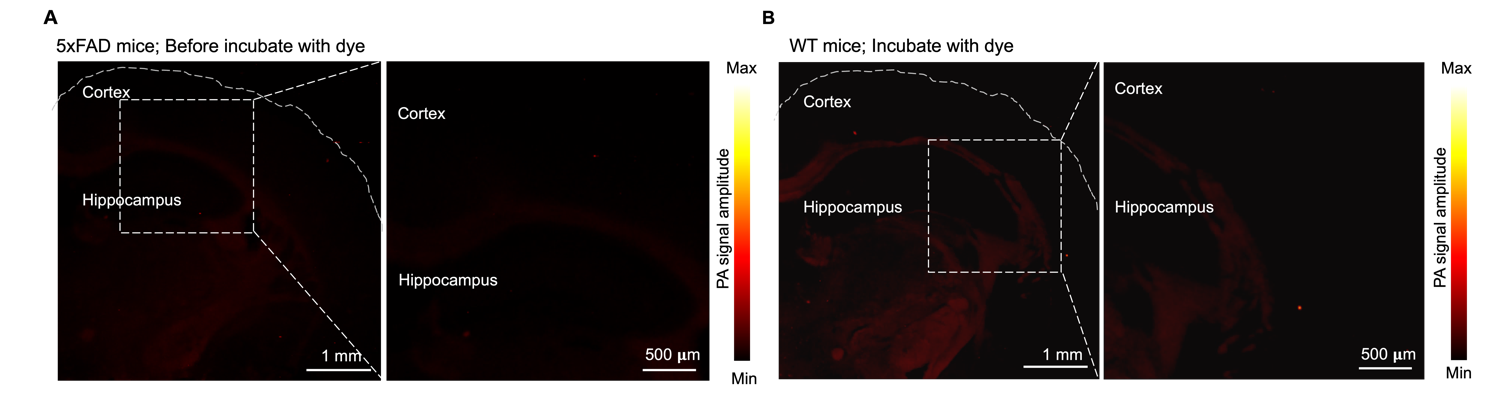
**

**Figure S1.** **Multispectral PAM imaging of Aβ plaques in coronal brain sections.**

(A) Baseline PAM image of a 5xFAD mouse brain section before AOI987 (Aβ-specific probe) incubation, showing intrinsic background signal. (B) PAM image of a WT mouse brain section after AOI987 incubation, demonstrating probe specificity for Aβ deposits.

Left panels: Full-section overviews (scale bar: 1 mm).

Right panels: Magnified ROIs highlighting hippocampal regions (scale bar: 500 μm).

Abbreviations: Ctx, cortex; Hp, hippocampus.


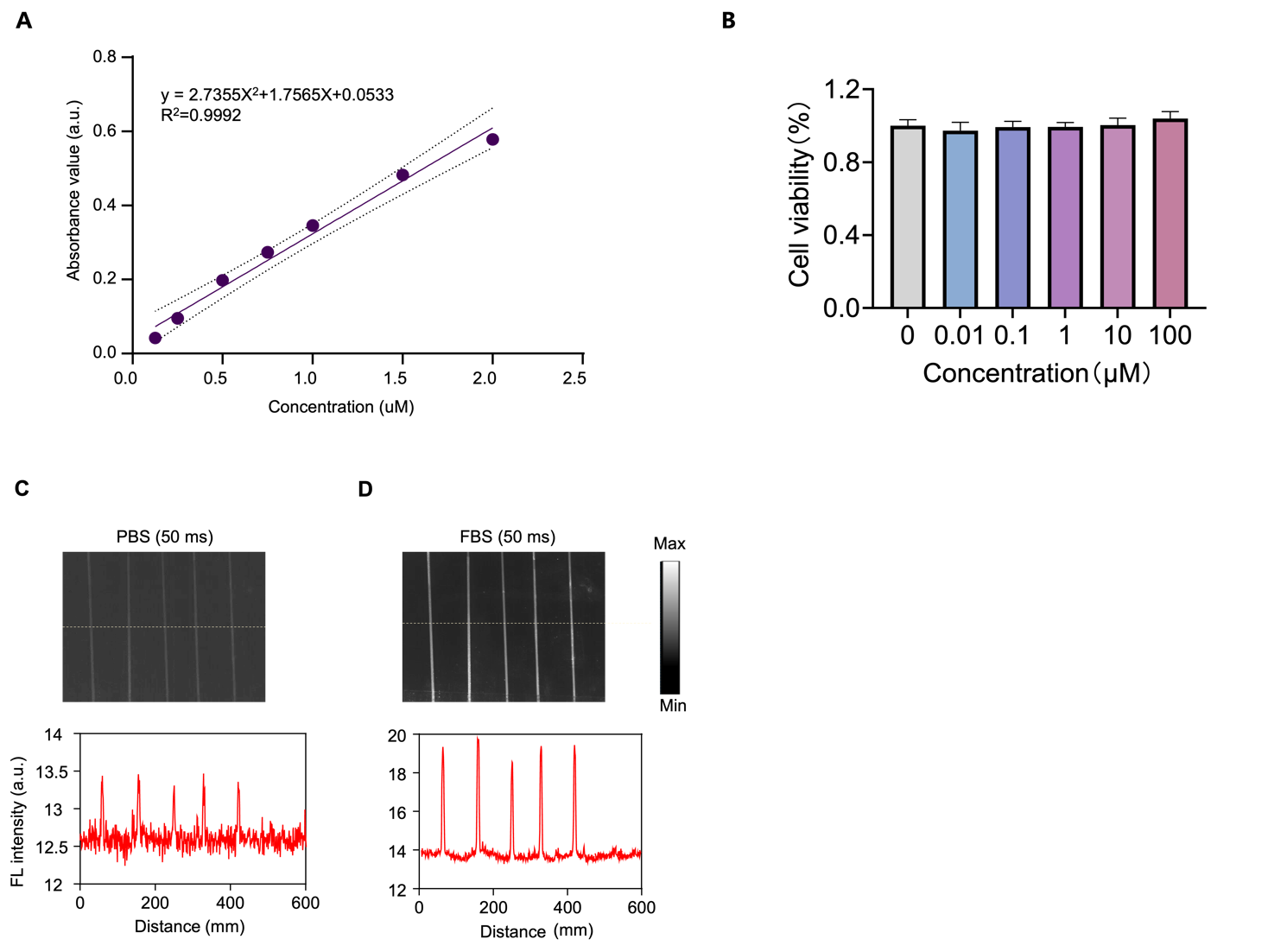


**Figure S2.** **Photophysical characterization of TREM2-ICG microglia-targeted probe.**

(A) Conjugation scheme of TREM2 antibody with ICG-NHS ester (method adapted from Ref 24). Inset: BSA analysis confirming conjugation efficiency. (B) Conjugation efficiency validation by BSA assay. Spectrophotometric quantification shows a linear correlation (R² = [0.992]) between TREM2-ICG probe concentration (0-100 μM) and absorbance at 280 nm (antibody) and 780 nm (ICG), confirming stoichiometric conjugation. (C, D) Environment-dependent fluorescence: NIR-II emission (>1000 nm) under 808-nm excitation. (C) PBS suspension exhibits minimal signal (SNR <2:1), while (D) equivalent concentration in 10% FBS shows 1.5-fold enhancement due to protein-binding stabilization.


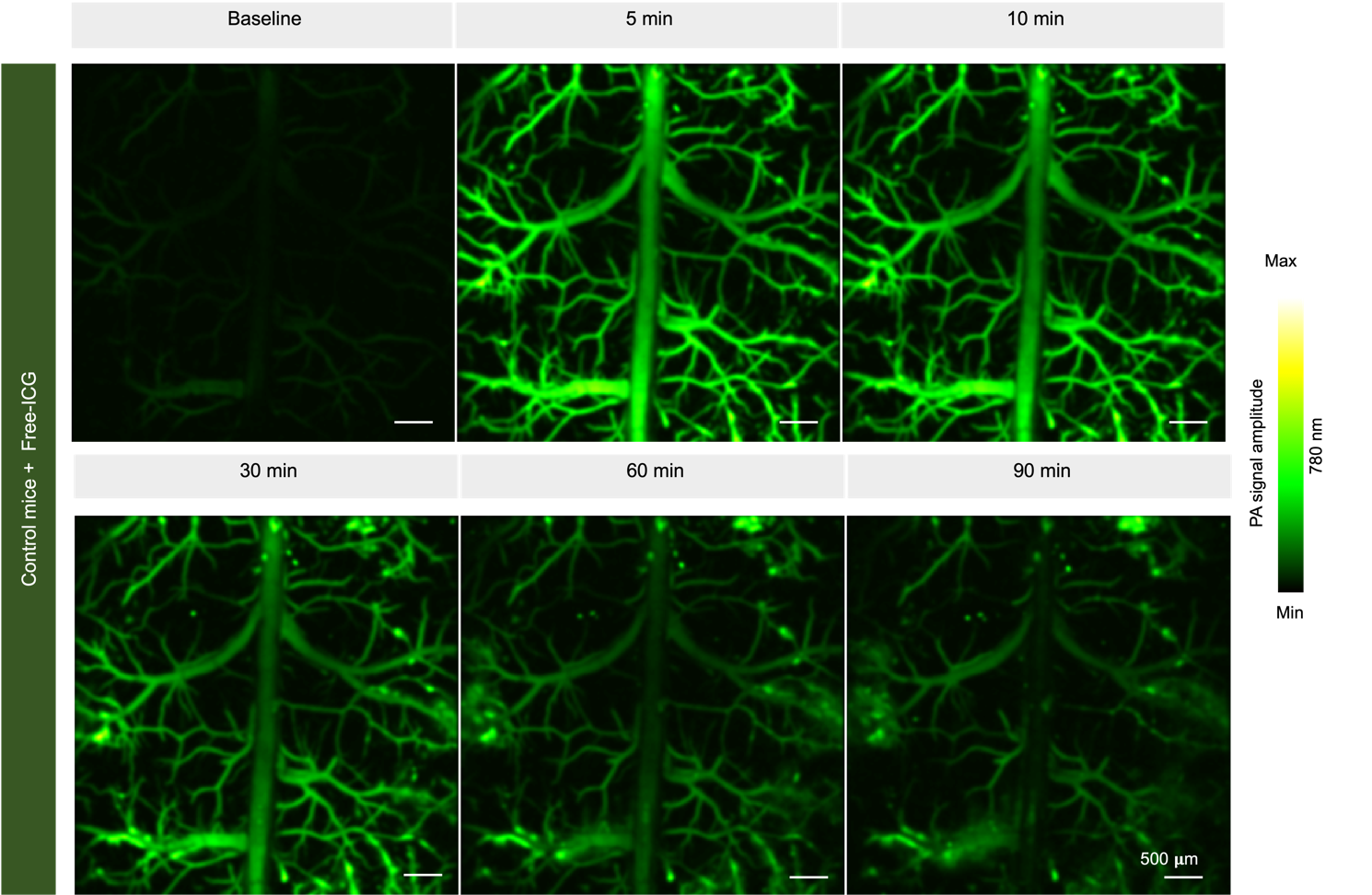


**Figure S3.** **Free ICG clearance kinetics.** A substantial signal decrease occurred in wild-type mouse brains by 30 min post-IV administration.


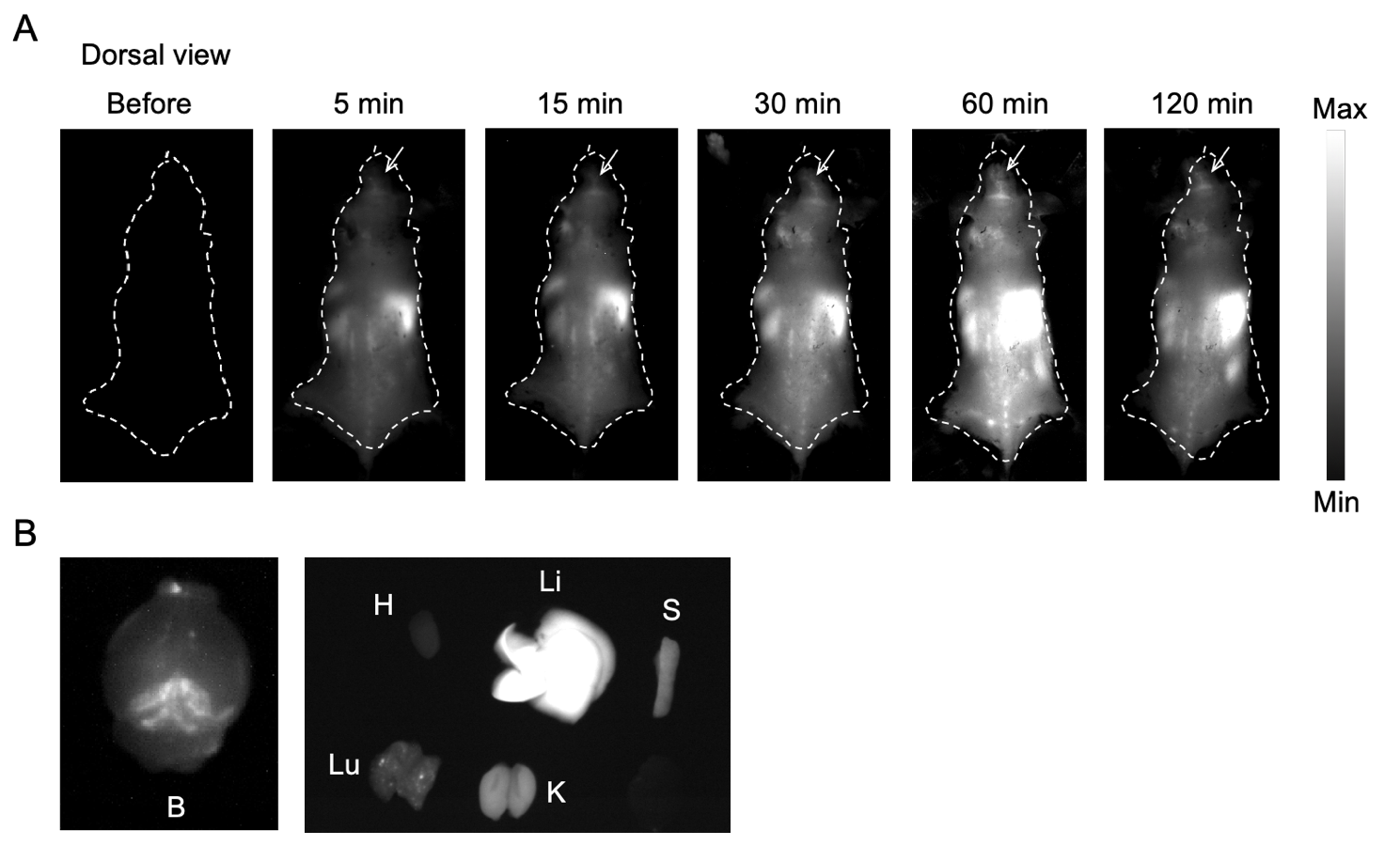


**Figure S4. Biodistribution of ICG-TREM2 in mice following intravenous injection.**

(A) Representative time-dependent *in vivo* NIR-II fluorescence images (dorsal view) of mice before and at 5, 15, 30, 60, and 120 min after injection. The white arrow highlights the accumulation of the probe in the brain region over time. (B) *Ex vivo* fluorescence images of major organs harvested at 120 min post-injection. The probe shows significant uptake in the target organ (brain) and the metabolic organ (liver), with lower accumulation in other tissues.

Abbreviations: B: Brain; H: Heart; Li: Liver; S: Spleen; Lu: Lungs; K: Kidneys.


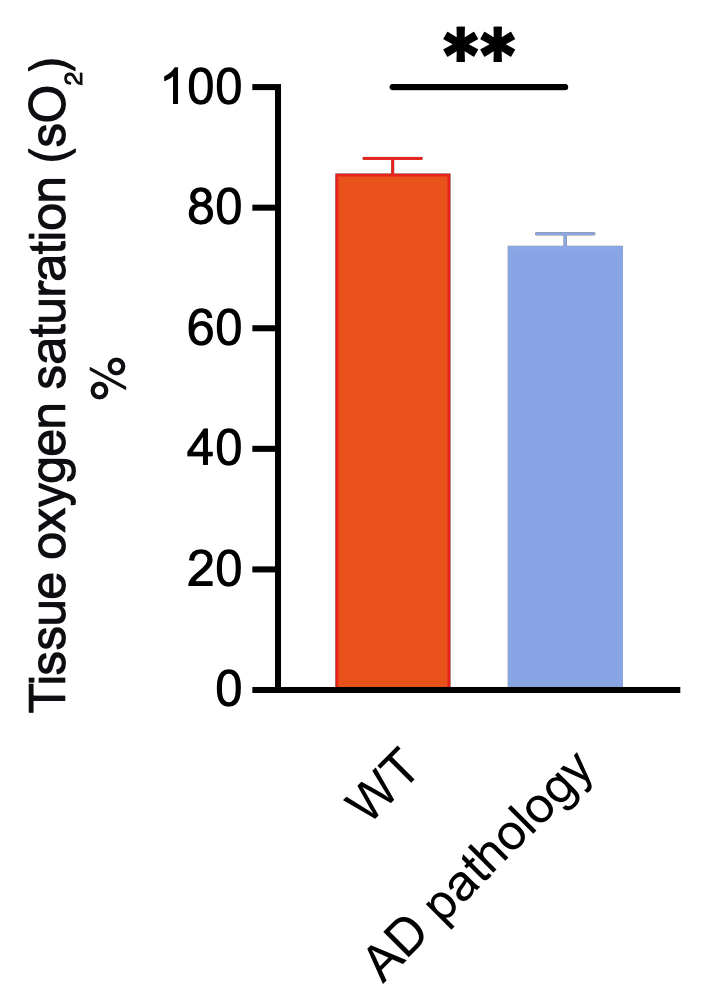


**Figure S5. Reduced Cerebral Oxygen Saturation in AD Mice.** Quantification of cerebral tissue oxygen saturation (sO₂). The bar graph compares the average sO₂ levels between WT controls and the AD pathology group. The AD group exhibits significantly reduced oxygen saturation compared to the WT group, indicating cerebral hypoxia (**P < 0.01). Data are presented as mean ± SD.
